# From Materials to Devices: Multiscale Modeling of Colloidal Quantum Dot Optoelectronics

**DOI:** 10.3390/nano16211338

**Published:** 2026-10-22

**Authors:** Phung Phuong Uyen, Byeongsu Kim

**Affiliations:** 1 Department of Electrical, Electronic and Computer Engineering, University of Ulsan, Ulsan 44610, Republic of Korea; phungphuonguyen1@gmail.com; 2 School of Electrical and Electronics Engineering, University of Ulsan, Ulsan 44610, Republic of Korea

**Keywords:** colloidal quantum dot, optoelectronic devices, quantum confinement, theoretical modeling, electron hopping, solar cells, photodetectors, light-emitting diodes

## Abstract

Colloidal quantum dots (CQDs) have emerged as a versatile platform for optoelectronics due to their solution processability, size-dependent tunable bandgaps and strong light-matter interaction. In this review, we systematically examine how theoretical models for CQD optoelectronic devices have been developed and applied. This review emphasizes the importance of integrating theoretical models across CQD materials and optoelectronic devices to enable accurate prediction of key optoelectronic performance. Recent advances in theoretical frameworks for CQD optoelectronics have enabled the prediction of material properties (electronic structure, defect formation, absorption and emission spectra) through atomic-scale simulations and device behaviors (charge separation, transport, recombination and multiplication) through device-scale simulations. These integrated approaches propose the design principles for developing the CQD architectures with optimized optoelectronic performances, enabling practical implementation in device fabrication. We conclude that the future incorporation of machine learning with theoretical CQD models ensures the exploration of previously inaccessible CQD materials and device architectures, accelerating the commercialization of CQD-based optoelectronic applications.

## 1. Introduction

As artificial intelligence continues to penetrate all aspects of our lives, growing emphasis has been placed on seamless interconnection and communication between humans and machines. This demand drives the need for high-performance optoelectronic devices including light-emitting diodes, solar cells and photodetectors. However, conventional optoelectronic devices containing silicon, germanium and compound semiconductors often require epitaxial growth and precise composition engineering to achieve high performances [1,2,3], leading to complex and expensive manufacturing.

Colloidal quantum dots (CQDs) are nanocrystalline semiconducting materials with dimensions smaller than their exciton Bohr radius [4,5], enabling strong quantum confinement effects. These characteristics make the CQDs a powerful platform for optoelectronic applications, offering facile bandgap tunability [6,7,8] and strong photon absorption and emission [9,10]. Moreover, the solution processability of CQDs provides a pathway for low-cost and scalable manufacturing through spin coating, blade coating, inkjet printing and roll-to-roll processing.

Recent advances in CQD chemistry have significantly improved the optical and electrical characteristics of CQD-based optoelectronic devices [11,12,13,14]. Specifically, the development of CQD inks employing short ionic ligands has enabled suppressed defect densities at CQD surfaces and efficient electron hopping between neighboring CQDs, achieving certified power conversion efficiency (PCE) exceeding 13% in CQD solar cells (QDSCs) [15,16,17]. The introduction of ligands that provide optimal dot-to-dot distances facilitates efficient charge multiplication within the CQD region, yielding superior detectivity beyond the theoretical limit in CQD photodetectors (QDPDs) [18,19]. In CQD light-emitting diodes (QLEDs), mixed ligand strategies that combine long- and short-chain ligands have emerged to balance surface passivation and charge injection, achieving superior electroluminescence capability [20,21].

Meanwhile, the development of theoretical CQD models is crucial for elucidating the underlying physical mechanisms, predicting experimental device performance and developing device architecture for efficient optoelectronic applications. Atomistic simulations employ computational approaches, including effective mass approximation (EMA), density functional theory (DFT) [9,22,23,24,25,26,27], time-dependent DFT (TD-DFT) [8,28], finite element methods [29], tight-binding [5] and Monte Carlo methods, to calculate the key CQD properties such as electronic structures, optical transitions, surface defects and carrier behaviors based on the atomic compositions and configurations. Technology computer-aided design (TCAD) platforms, including Synopsys Sentaurus, Silvaco ATLAS and the open-source SCAPS-1D tool, have become widely adopted to design the architecture and estimate carrier dynamics, energy band alignment and device performance in CQD-based optoelectronic devices [30,31,32,33,34]. Moreover, the transfer matrix method (TMM) [35] and finite-difference time-domain (FDTD) [36] simulations enable the analysis of electromagnetic field distributions and optical absorption characteristics within specific CQD regions.

Despite this progress, these tools are still frequently applied in isolation. Recent efforts have begun to bridge this gap by linking DFT, device-level simulators (e.g., SCAPS), Lambert W-based parameter extraction and machine learning into integrated computational pipelines for quantum dot solar cells [37]. However, such approaches are generally formulated for QD material systems broadly and are centered on a single device application. A dedicated survey of how multiscale models are applied to CQDs across their optoelectronic applications is still lacking.

In this review, we systematically investigate recent advances in theoretical models for CQD-based optoelectronic devices, covering computational approaches from CQD materials to complete device architectures. In Section 2, we survey basic principles and simulation frameworks of atomic-scale CQDs. Section 3 addresses theoretical approaches to modeling CQD surface chemistry, including ligand-physics frameworks, the atomistic origin of trap states and strategies for their theoretical elimination, hopping-based charge transport and charge recombination in CQD solids, and the environmentally induced degradation of CQD films together with theoretical strategies to mitigate it. Section 4 elucidates the operating principles and simulation frameworks for CQD-based optoelectronic devices, with a focus on their physics and performance optimization. Finally, we highlight the future perspectives on integrating atomistic modeling, device simulation and machine learning to enable the predictive discovery of novel CQD materials and advanced optoelectronic devices in Section 5.

## 2. Theoretical Frameworks for Electronic Structure of CQDs

In most CQD-based optoelectronics, the CQD layers play a critical role in the overall device architecture, enabling photon absorption in QDPDs and QDSCs [34,38,39,40,41] and photon emission in QLEDs [42,43]. In QDPDs and QDSCs, incident photons are absorbed within the CQD layer to generate electron-hole pairs, whose subsequent transport, recombination and extraction determine device performance. In QLEDs, however, electrons and holes are electrically injected from opposite electrodes into the CQD emissive layer, where they recombine radiatively to generate photons. This suggests that a comprehensive understanding of how the quantum confinement effect influences the optical properties of the CQD ensemble is essential. This section reviews the key theoretical frameworks developed to describe the electronic structure and optical absorption of CQDs, ranging from simple analytic models to atomistic approaches that reflect the complexity of realistic nanocrystal systems.

The basic theoretical framework for considering quantum confinement in CQDs is the effective mass approximation (EMA) [44]. The EMA is based on expanding the electron (or hole) wavefunction in terms of Bloch functions and adopting the parabolic approximation for the full band dispersion, *E*(*k*) ≈ *ħ*^2^*k*^2^/2m*, near the band edge, where the effective mass (m*) is determined by the curvature of the band edge [45].

The EMA provides a simple framework for describing carrier confinement in CQDs by treating carriers as quasiparticles with bulk effective masses and slowly varying envelope wavefunctions. In its conventional form, EMA is often combined with a spherical quantum well and an infinite barrier at the CQD boundary. This approach can capture the size-dependent quantization of carrier energy levels and provide useful estimates of confinement energies and optical transition energies [46].

However, its accuracy decreases as the CQD size approaches the nanoscale. Two limitations should be distinguished. First, the envelope-function and parabolic-band approximations become less reliable under strong confinement, where the envelope varies on the scale of the crystal lattice and carriers sample regions far from the band edge. Multiband *k·p* models can partially address these effects while retaining the effective-mass framework [47]. Second, the commonly assumed infinite spherical barrier is an idealized confinement model rather than an intrinsic requirement of EMA. Finite barriers, realistic CQD shapes and surface or ligand potentials can instead be incorporated to describe wavefunction leakage and surface-related effects more realistically. Thus, EMA is particularly useful for understanding the basic size-dependent quantum confinement of CQDs, but becomes insufficient for describing atomistic surface structure, localized trap states and other strongly surface-dependent phenomena [28], as discussed in the following section.

Alternatively, first-principles calculations based on density functional theory (DFT) offer an atomistic description of CQDs that captures quantum mechanical interactions among constituent atoms and enables accurate prediction of their electronic structures and optical properties beyond the EMA. Hohenberg and Kohn demonstrated that DFT reformulates the many-body electron problem by replacing individual electron wavefunctions with electron density, thereby providing a computationally tractable route for determining ground-state electronic structure with minimal reliance on empirical parameters [48]. This methodology enables the DFT calculations to explicitly incorporate the atomic compositions and dimensions of CQD constituents, including core, shell and ligands together with defect configurations, thereby providing the electronic structures of actual CQD conditions [49]. In a well-defined DFT model, CQD surfaces require no passivation with artificial hydrogen-like pseudo-ligands [50,51], while geometry optimization using realistic ligands without ad hoc parametrization yields an accurate description of quantum-confined energy levels and surface states [6]. Accurate surface stoichiometry is essential in DFT models, as disproportionate numbers of metal and nonmetal surface ions cause artificial midgap trap states or unintended doping, significantly distorting the electronic structure relative to actual CQDs [6].

Even with a properly constructed structural model, the accuracy of DFT depends on the choice of exchange-correlation functional. Standard DFT calculations employing semi-local exchange-correlation functionals (e.g., LDA, GGA) suffer from a self-interaction error, which leads to a systematic underestimation of the band gap and can cause defect-state energy levels to be mislocalized or artificially delocalized near the valence band edge, thereby distorting the predicted electronic structure of CQDs [52,53]. Several strategies have been developed to mitigate these limitations. Hybrid functionals such as HSE06, which incorporate a fraction of exact Hartree-Fock exchange to partially cancel the self-interaction error, have been shown to substantially improve band-gap accuracy relative to GGA for both lead-chalcogenide and II-VI CQD systems, bringing calculated values into good agreement with experiment [54,55,56,57]. The GW approximation instead computes quasiparticle energies from many-body perturbation theory, providing a more rigorous description of the band gap and defect-state positions, though at substantially higher computational expense that limits its application to relatively small CQD systems [58]. For systems containing localized or strongly correlated states, such as certain dopant or defect-derived orbitals, DFT + U (Hubbard-type) corrections introduce an on-site Coulomb repulsion term to correct the self-interaction error associated with these localized states [59,60]. While these approaches improve the fidelity of material-scale electronic structure predictions, their increased computational cost remains a key consideration when extending such calculations toward device-scale modeling [25].

Moreover, time-dependent DFT (TDDFT) calculations extend the conventional DFT to excitation behavior, enabling the prediction of optoelectronic properties, such as the absorption spectra, exciton-phonon interaction and charge distribution in excited states [61]. TDDFT reliably estimates optical transitions at low-energy states in response to surface stoichiometry and ligand modifications, making it a valuable tool for guiding surface engineering strategies. Unfortunately, TDDFT struggles to describe highly correlated electronic states, including biexcitons and states with strong double excitation character because of the approximations inherent in most practical TDDFT calculations [28,62]. Several strategies have been developed within the TDDFT framework to address this limitation. Dressed TDDFT introduces a frequency-dependent correction to the adiabatic kernel for Kohn-Sham single excitations that lie close in energy to a double excitation, enabling their mixing and reproducing the correct excitation energies of the interacting system [62]. Spin-flip TDDFT instead reformulates the reference state used for the linear-response calculation by choosing a high-spin triplet reference, double excitations with respect to the closed-shell ground state appear as single excitations of the new reference, making them accessible via standard adiabatic kernels [63,64]. The particle-particle random phase approximation (pp-RPA), which uses an (N-2)-electron reference state, provides a related but distinct route in which double excitations arise naturally from the simultaneous addition of two electrons and has also proven effective for charge-transfer excitations [65]. However, each of these approaches carries practical limitations for CQD systems. In particular, dressed TDDFT requires manual identification of the relevant Kohn-Sham excitations and breaks down precisely in the regime of near-degenerate band-edge states characteristic of quantum-confined systems, while pp-RPA does not reliably capture excitations with substantial hole character beyond the highest occupied level, which is a common feature of CQD valence-band structure. Moreover, none of these methods has yet been demonstrated on realistically sized, ligand-passivated CQDs with explicit atomistic structure, making these approaches better suited, at present, to molecular or model systems than to the atomistic, surface-dominated electronic structure of CQDs. In this sense, the introduction of non-adiabatic dynamics (NAD) approaches, which combine TDDFT with surface hopping techniques, enables the prediction of ultrafast excited-state carrier dynamics, including carrier relaxation, electron-phonon interaction and non-radiative energy dissipation, thereby verifying the influence of surface ligands on these processes [61,66].

Semiempirical models including tight-binding [5,28,67,68] and empirical pseudopotential [8,69] methods provide a practical balance between the computational cost and the atomistic accuracy. In the tight-binding approach, the electronic structure is constructed from localized atomic orbitals, while the Hamiltonian is parameterized using on-site energies and interatomic hopping integrals that describe the interactions between neighboring atoms. This enables efficient calculations of large CQDs with atomistic information preserved. In contrast, the empirical pseudopotential method represents the crystal potential as a superposition of empirically fitted atomic pseudopotentials, allowing precise calculations of band structures, electronic states and optical transitions without explicitly treating core electrons. In these frameworks, the atomic structure of CQDs is described using parametrized Hamiltonians, while the surface ligands are represented by effective parametrized potentials [28]. However, the accuracy of these approaches strongly depends on their empirical parameters, limiting their applicability to CQDs with different ligand species, compositions and structural configurations [28].

**Figure 1:**
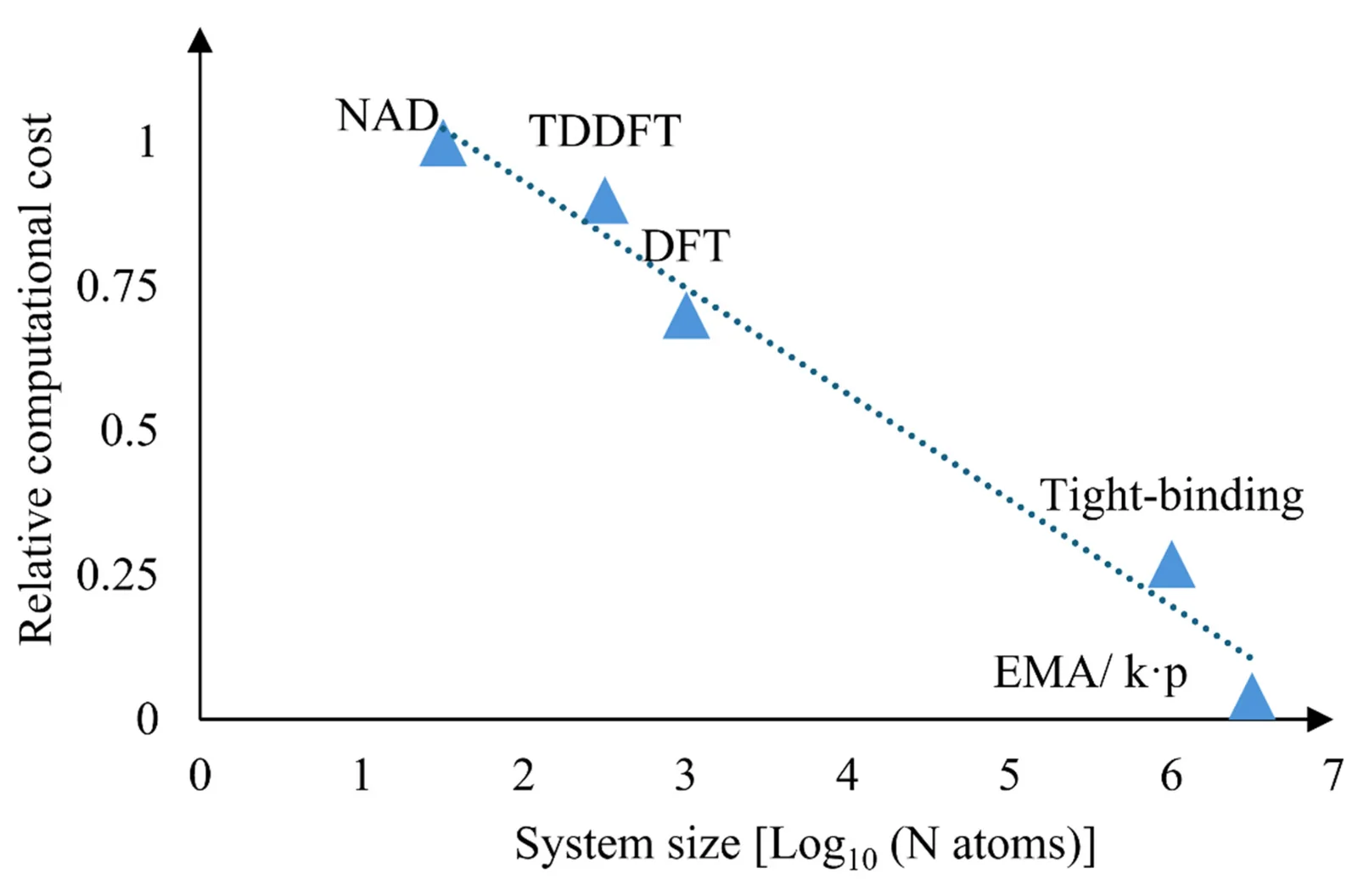
Relative computational cost as a function of system size for atomic-scale theoretical simulations based on EMA, tight-binding, DFT, TDDFT and NAD [70,71,72,73,74,75].

Conventionally, higher accuracy for realistic models inevitably requires greater computational cost and restricts the accessible system size (Figure 1). The EMA is the least computationally demanding method, enabling simulations of systems containing millions of atoms. However, an overly simplified description of the electronic structure limits predictive accuracy. In particular, EMA is unsuitable for CQD modeling due to its approximate treatment of surface states, ligand chemistry and atomic-scale structural variations, which critically influence the CQD properties. Conversely, atomistic methods such as DFT and NAD provide a detailed description of CQDs by explicitly accounting for atomic structure and electronic interactions. This improved accuracy, however, comes at the expense of substantially higher computational cost, restricting simulations to much smaller systems. Despite these limitations, DFT and NAD remain widely employed computational approaches in CQD research because they enable accurate investigation of electronic structure, carrier dynamics, surface chemistry and excited-state processes.

Although these theoretical frameworks discussed above provide a fundamental understanding of the electronic structures and optical properties of individual CQDs, actual CQD optoelectronic devices operate as thin films rather than isolated particles. In CQD thin films, adjacent CQDs become electronically coupled, while ligands and defects at CQD surfaces collectively determine the strength of electronic coupling, carrier transport and trap density. Consequently, a comprehensive understanding of CQD optoelectronics requires extending the scope from the electronic structure of the isolated CQDs to the electronically coupled CQD films, where surface chemistry governs electronic coupling, charge transport and defect formation. Section 3 therefore discusses the fundamental principles of carrier dynamics in CQD thin films, beginning with how surface ligands and surface modification influence electronic coupling and charge transport, followed by charge recombination and trap-mediated losses, and concluding with how environmental factors degrade the surface and how this degradation can be mitigated.

## 3. Fundamental Principles for Carrier Dynamics in CQD Films

Understanding carrier dynamics in CQD thin films is essential for developing theoretical models of high-performance CQD optoelectronic devices. Notably, the surface characteristics, determined by the types of semiconducting CQDs and capping ligands, process methods of ligand exchange and defect formation strongly influence charge transport and recombination within the CQD region. The same surface characteristics also govern the response of CQD films to environmental stressors such as moisture, oxygen, illumination and heat, which generate traps and ultimately limit device stability. This section discusses surface modification strategies in CQD films for improving the performance of optoelectronic devices, along with their influence on charge transport and recombination. It then summarizes the theoretical mechanisms by which environmental factors degrade CQD films and the simulation-guided strategies that have been used to improve stability.

### 3.1. Surface Modification of CQD Thin Film for Optoelectronic Devices

Despite the remarkable optoelectronic properties arising from quantum confinement in individual CQDs, the device performances are strongly influenced by their surface characteristics. Particularly, sequential deposition of CQD films inevitably induces incomplete passivation, followed by oxidation and CQD aggregation, causing the formation of electronic trap states.

Trap states in CQDs are localized electronic states with energy levels lying within the bandgap between the valence band maximum (VBM) and conduction band minimum (CBM) [27]. This is primarily attributed to incompletely coordinated surface atoms, which are widely recognized as the principal structural source of trap states [76,77,78,79]. Notably, these defect states at CQD surfaces increase with diminishing CQD diameters [80]. Unlike atoms in the bulk region of CQDs, surface atoms are undercoordinated, leading to spatially localized electronic states and reactive sites that are susceptible to chemical transformations [26]. These undercoordinated atoms tend to leave unsaturated valence orbitals, disrupting the periodic electronic potential of the CQD lattice. Hence, the unsaturated valence orbitals introduce localized electronic states within the bandgap that act as trap states, promoting trap-assisted non-radiative recombination [81]. For example, Se atoms at surfaces with only two Cd neighbors are primarily responsible for the formation of localized trap states in CdSe CQDs, causing hole localization and subsequent non-radiative recombination [77,82,83]. In CdS CQDs, sulfur vacancies at the CQD surfaces cause the undercoordination of adjacent Cd atoms, introducing localized trap states within the bandgap [80]. In addition to intrinsic vacancies and undercoordinated surface atoms, surface oxidation is another key mechanism for generating trap states. Oxidized chalcogen species (e.g., Se-O or S-O) alter the local bonding environment, introducing localized electronic states within the bandgap that capture carriers and facilitate non-radiative recombination, thereby degrading the optical performance and stability of CQDs [26].

These trap states drastically degrade the performance in CQD-based optoelectronic devices. Deep trap states, which are located near the center of the CQD bandgap, capture the electrons and holes. These captured electron-hole pairs tend to recombine, thereby reducing carrier mobility and shortening carrier lifetimes [81]. The reduction in carrier mobility disturbs the extraction of photogenerated carriers, thereby limiting the PCE in QDSCs and the detectivity (D*) in QDPDs. Moreover, this recombination releases the energy through the emission of multiple phonons, having energies lower than the CQD bandgap, leading to the reduction in photoluminescence quantum yields (PLQY)s in QLEDs [84]. Meanwhile, shallow traps are defect-induced electronic states located close to the conduction or valence band edges of CQDs [26]. Unlike deep traps, carriers captured by shallow traps undergo trapping-detrapping processes because their activation energy facilitates thermal re-emission of captured carriers, rather than causing permanent carrier loss. In QDPDs, these trapping-detrapping phenomena in shallow trap states drastically increase the flicker noise current and reduce the response time, thereby reducing the D* [85].

**Figure 2:**
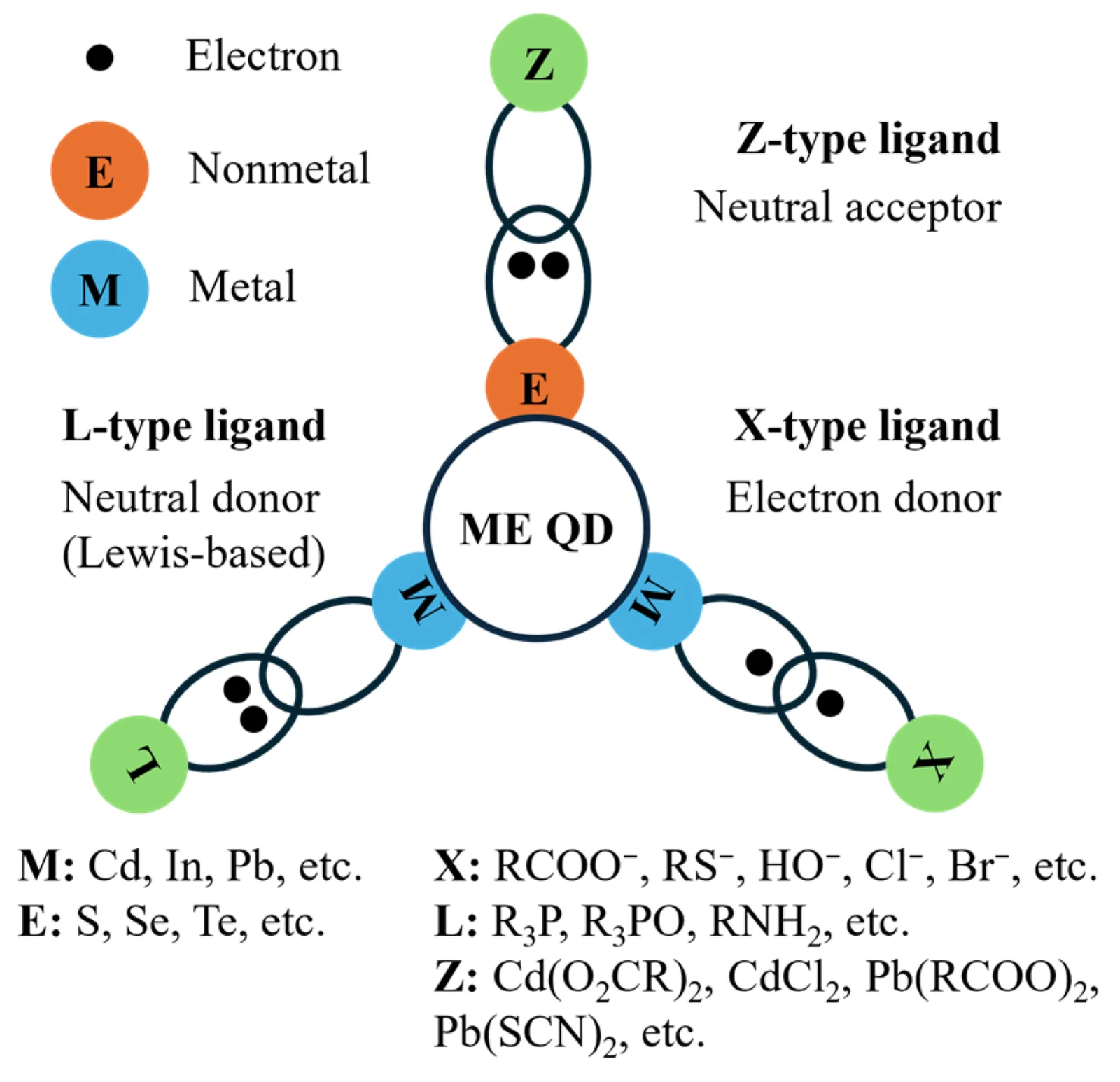
Schematic illustration of X-, L- and Z-type ligand binding motifs on CQDs. X-type ligands are anionic, single-electron donors that passivate exposed metal cation sites, whereas L-type and Z-type ligands act as neutral donors and neutral acceptors, respectively.

In this sense, the design of ligands capped by CQD surfaces is crucial because the optoelectronic characteristics of the semiconducting core and the encapsulating ligands are intrinsically coupled [6]. Ligands are essential not only for providing colloidal stability but for regulating nanocrystal growth during synthesis, passivating CQD surfaces and tuning the electronic structure of CQDs [86,87,88]. These ligands are typically classified into X-type, L-type and Z-type based on the number of electrons they donate to or accept from the surface metal coordination sites (Figure 2) [12,13]. X-type ligands are anionic species that donate a single electron to uncoordinated surface metal cations, thereby passivating positively charged surface sites through strong coordinative bonding [13]. L-type ligands are electrically neutral Lewis bases that coordinate to surface metal atoms through a lone electron pair. They effectively passivate uncoordinated surface atoms without altering the net charge of the nanocrystal, although their binding strength is considerably weaker than that of X-type species [13]. Z-type ligands are neutral electron-accepting (Lewis acidic) moieties that selectively bind to surface chalcogen anions, passivating anionic surface defects and improving both the PLQY and long-term colloidal stability [12].

Long-chain organic ligands are employed to regulate nanocrystal nucleation and growth while maintaining colloidal dispersibility for CQD synthesis [12,87]. However, their insulating nature and steric hindrance render efficient charge transport difficult in CQD solids. Moreover, the residual long-chain ligands significantly impede complete surface passivation, creating electronic trap states that reduce QYs in CQDs and PCEs in QDSCs [89,90]. Consequently, ligand exchange has become an indispensable processing step for developing high-performance CQD optoelectronic devices [91,92,93]. Depending on the CQD phase, ligand exchange is classified into solid-state and solution-state exchange. In solid-state exchange, the CQD layers are sequentially deposited and treated with shorter ligands to replace the original long-chain organic ligands after the deposition step [88]. This replacement reduces the dot-to-dot distances between adjacent CQDs and enhances charge hopping probability by narrowing the ligand-mediated potential barriers [39,94]. Unfortunately, sequential deposition of CQD films for a thick absorption layer would cause solvent penetration, resulting in ligand detachment on the previously deposited CQD layer, as well as an expensive process with excessive time consumption and material waste [95,96]. Solution-phase exchange overcomes these limitations by performing ligand substitution while CQDs remain colloidally dispersed, typically through phase transfer from a nonpolar to a polar solvent, enabling more complete ligand displacement and superior characteristics for surface modification [94]. Owing to its high exchange efficiency and enhanced tunability of surface stoichiometry, solution-phase ligand exchange has emerged as the predominant strategy for CQD device fabrication [93].

The replacement of native long-chain ligands with short ligands, particularly ionic and thiol ligands, provides several advantages for improving optoelectronic performance and stability in CQD devices. First, the compact molecular dimensions of short ligands compared with long-chain ligands enable denser packing and effective ligand coverage of CQDs. This compact ligand coverage at CQD surfaces not only reduces steric hindrance between adjacent CQDs [92] but also establishes a chemically robust passivation layer that suppresses the penetration of oxygen species, both of which intensify degradation in CQD films [94]. Next, short ligands effectively passivate undercoordinated surface atoms that would otherwise introduce localized electronic states within the forbidden gap. The elimination of these midgap trap states suppresses trap-assisted non-radiative recombination [97,98], thereby extending carrier lifetime [91] and elevating the radiative recombination fraction [94]. Third, the shortened ligand length decreases the dot-to-dot distances within the CQD solid, facilitating electron hopping between neighboring CQDs, which is essential for efficient charge collection in CQD optoelectronic devices. It is worth noting that shortening the ligand length compromises the steric stabilization, allowing attractive interactions between CQDs to exacerbate CQD aggregation during film deposition. This results in morphological inhomogeneity and nonuniform electronic coupling within CQD solids, thereby offsetting the transport benefits and ultimately restricting performance in CQD optoelectronic devices [99].

Unlike unintentionally introduced trap states, intentional doping introduces impurity atoms into the CQD lattice by design, giving rise to dopant-derived electronic states whose energy and degree of localization depend on the identity, concentration and lattice position of the dopant. Doping is among the most common and most extensively studied sources of midgap states in CQDs [26]. The resulting dopant-induced electronic states are experimentally and spectroscopically distinct from surface trap states. For example, in Bi-doped PbS CQDs, substitutional n-type doping introduces discrete dopant-induced states approximately 0.3–0.35 eV below the conduction band edge, which give rise to characteristic donor-bound exciton recombination and donor-to-valence-band radiative transitions with distinct temperature-activated trapping/detrapping dynamics that depend on whether doping occurs during synthesis or post-synthetically via cation exchange [100]. Such dopant-induced states can be deliberately exploited to engineer desirable optoelectronic functionality including tunable sub-bandgap absorption/emission, controlled carrier density and magneto-optical coupling in transition-metal-doped systems [100,101].

### 3.2. Charge Transport Mechanism in CQD Solids

Charge transport in CQD films is governed by the electronic landscape of the nanocrystal solid: individual CQDs possess discrete energy levels, while surface ligands create potential barriers between adjacent CQDs that form semiconductor–insulator–semiconductor junctions. Unlike bulk semiconductors with continuous energy bands, charge carriers in CQD solids migrate through sequential hopping between localized states of adjacent CQDs by overcoming potential barriers mediated by ligands. This hopping probability is therefore determined by electronic coupling between adjacent CQDs, which is strongly influenced by the dot-to-dot distances.

The carrier transport in CQD solids is described by a thermally activated hopping model, in which carrier mobility is determined by the hopping probability and the average distance between adjacent CQDs [6]. The hopping mobility in three dimensions is derived from the Einstein relation between mobility and diffusion *μ* = *eD*/*k_B_T*, where *D* = *d*^2^/(6*τ_hop_*) is the diffusion coefficient. Therefore, mobility in this case is expressed as:

$$\mu =\frac{ed^{2}}{6\tau_{hop}k_{B}T}$$

where *e* is the elementary charge, *d* is the dot-to-dot distance between neighboring CQDs, *τ_hop_* is the effective hopping time between adjacent dots, *k_B_* is the Boltzmann constant and *T* is the absolute temperature [102]. Here, hopping time *τ_hop_* typically follows an Arrhenius form *τ_hop_* = *τ*_0_ *exp*(*E_a_*/*k_B_T*), where *E_a_* is the activation energy carriers must overcome to hop to a neighboring site and *τ*_0_ is a temperature-independent factor related to the electronic coupling strength and attempt frequency. As the length of surface ligands becomes shorter, *d* decreases, while dot-to-dot coupling is strengthened, leading to an exponential increase in the hopping probability and substantially shortening *τ_hop_*. Consequently, the reduction in *τ_hop_* outweighs the decrease in *d*^2^ [103]; shorter ligands generally yield substantially higher mobility overall.

Under the standard Arrhenius NNH picture described above, mobility decreases as temperature decreases, since carriers have progressively less thermal energy available to surmount the activation barrier *E_a_*. An ideal band-like transport exhibits decreasing mobility with increasing temperature because of intensified phonon scattering at higher temperatures, providing an experimental signature that distinguishes it from hopping transport. However, thermally activated hopping is suppressed and the transport mechanism transitions to the variable range hopping regime at low temperatures, similar to that in disordered semiconductors [102].

The mobility required for efficient CQD optoelectronic devices is ultimately determined by their operating mechanisms. For QLEDs, modest mobility is sufficient because charge carriers only need to be transported across the emissive layer for radiative recombination [6]. Whereas high mobilities are required to ensure that the efficient *τ_hop_* is sufficiently shorter than the carrier radiative lifetime in the visible and near-infrared CQD solar cells and photodetectors. Because the radiative lifetimes range from tens of nanoseconds for CdSe CQDs to the microsecond regime for PbSe CQDs, mobilities of ~10^−2^ cm^2^/Vs are sufficient to facilitate efficient carrier hopping before recombination, thereby yielding ionization efficiencies above 99% [102]. Mid-infrared applications demand higher mobilities exceeding ~0.1 cm^2^/Vs because energy relaxation through ligand vibration and lattice phonons occurs on sub-nanosecond scales [104]. Moreover, faster non-radiative channels such as Auger recombination or exciton cooling further reduce the carrier lifetime, increasing the required mobilities for efficient CQD optoelectronic devices [102].

### 3.3. Charge Recombination and Trap-Mediated Losses in CQD Solids

Carrier recombination in CQD solids is rarely dominated by radiative recombination processes alone; instead, non-radiative pathways arise from trap states generated through surface defect density, incomplete ligand passivation, stoichiometric imbalances and CQD aggregation. Deep traps located near the middle of the bandgap strongly capture carriers and prolong their residence time, thereby exacerbating non-radiative recombination. Otherwise, shallow traps located close to the band edges temporarily localize carriers, disrupting dot-to-dot hopping and reducing the effective mobility [81].

**Figure 3:**
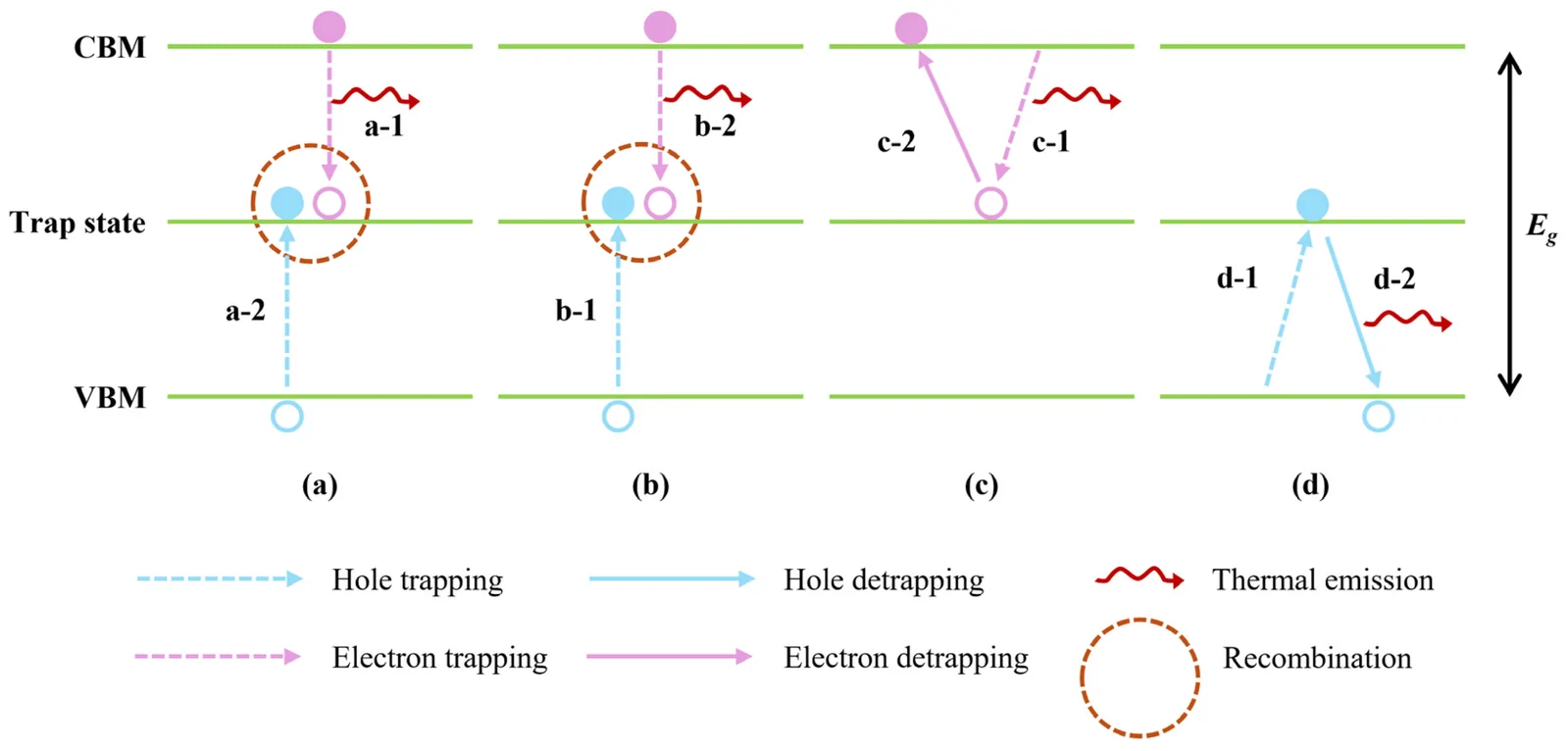
Schematic illustration of the four carrier transition pathways in the Shockley-Read-Hall model at a discrete trap state: (**a**) electron capture by an unoccupied trap state followed by recombination with a captured hole; (**b**) hole capture by an occupied trap state followed by recombination with a captured electron; (**c**) electron trapping followed by detrapping through thermal re-emission to the conduction band; and (**d**) hole trapping followed by detrapping through thermal re-emission to the valence band.

**Figure 4:**
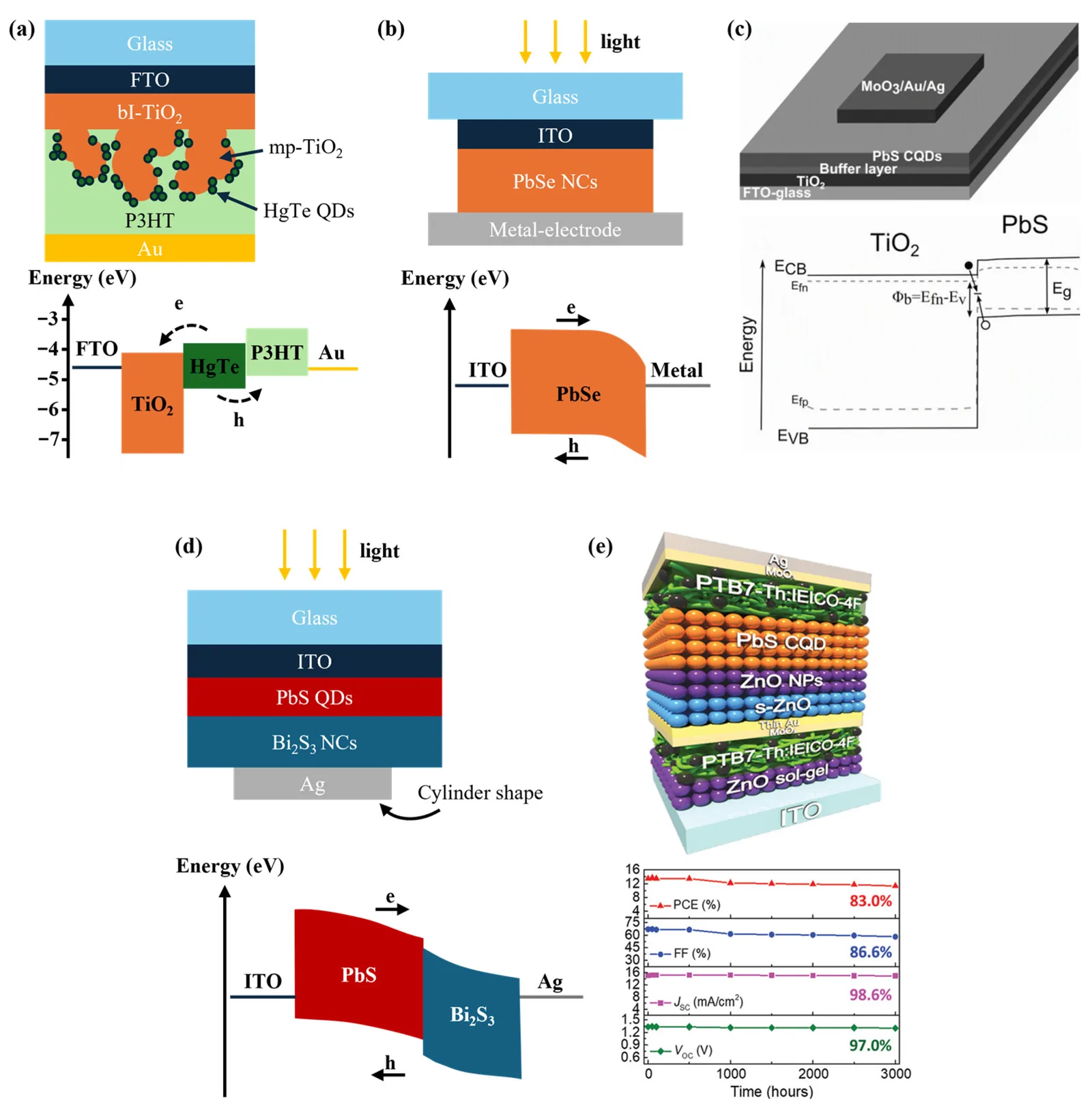
Representative device architectures of CQD-based solar cells. (**a**) Schematic illustration and band diagram of a QD-sensitized QDSC [125]. (**b**) Schematic illustration and corresponding band energy diagram of a Schottky junction QDSC [126]. (**c**) Schematic illustration and corresponding band energy diagram of a depleted heterojunction QDSC. Reproduced with permission [116]. Copyright 2013 John Wiley and Sons. (**d**) Schematic illustration and corresponding band energy diagram of a planar heterojunction QDSC consisting of ITO/PbS CQD/Bi_2_S_3_ nanocrystals/Ag [127]. (**e**) Schematic illustration and stability of an organic/PbS CQD hybrid tandem solar cell under ambient conditions without encapsulation. Reproduced with permission [128]. Copyright 2020 John Wiley and Sons.

The Shockley-Read-Hall (SRH) model formalizes the trap-assisted recombination through four carrier transition pathways at a discrete trap state [105,106] including: (1) electron capture by an unoccupied trap state followed by recombination with a captured hole (Figure 3a), (2) hole capture by an occupied trap state followed by recombination with a captured electron (Figure 3b), (3) electron capture (electron trapping) followed by thermal re-emission to the conduction band (electron detrapping, Figure 3c) and (4) hole capture (hole trapping) followed by thermal re-emission to the valance band (hole detrapping, Figure 3d).

Pathways (1) and (2) lead to net recombination, whereas pathways (3) and (4) represent temporary trapping with eventual carrier recovery. The balance between these outcomes is governed by trap density, trap energy depth, carrier capture cross-sections and the relative thermal escape rates [105,106]. Notably, the recombination rate at defect sites scales with carrier mobility [102], meaning that transport efficiency and recombination losses are highly correlated, as both are jointly rooted in the same surface chemistry and film morphology. This reinforces the conclusion from Section 2 that improved surface passivation directly enhances transport while suppressing recombination.

Beyond trap-mediated losses, multi-carrier interactions introduce intrinsic non-radiative channels independent of structural defects. When a CQD carries an excess charge, whether from intentional doping, carrier injection or trapping of one carrier type, the extra carrier receives the energy released by exciton recombination through a non-radiative Auger process. This process substantially quenches photoluminescence since the Auger recombination rate is orders of magnitude higher than the radiative rate [107]. This mechanism directly underlies photoluminescence blinking [108] and degradation [109] in nanocrystal films, reduced quantum efficiency in QLEDs [67,110] and elevated stimulated emission thresholds in CQD lasers [111].

The Auger recombination rate is given by Fermi’s Golden Rule [112]:

$$\frac{1}{\tau_{A}}=\frac{2\pi }{\hbar }\int {\left|M_{if}\right|}^{2}\delta \left(E_{i}-E_{f}\right)dR_{f}$$

where *M_if_* is the matrix element of the interparticle Coulomb interaction, *E_i_*_(*f*)_ is the initial (final) system energy and *R_f_* denotes the complete set of final state variables. Several features of this process are unique to the quantum-confined regime. Unlike bulk semiconductors, a carrier with kinetic energy comparable to the energy gap is unnecessary for Auger processes in confined structures [109]. The Auger lifetime is strongly size-dependent [113] and falls within the sub-nanosecond range for both charged excitons and biexcitons, far shorter than the tens-of-nanoseconds radiative lifetime of the neutral single exciton [114].

From a device engineering perspective, softening the confinement potential suppresses Auger recombination by reducing the Coulomb coupling matrix element *M_if_*. This approach has been proposed as a route to low-threshold CQD lasers and high-quantum-yield LEDs [115]. Because Auger recombination is strongly facilitated in charged excitons, surface passivation strategies that reduce unintentional charging provide a complementary route to suppressing this loss channel. Combining these offers a design rule for suppressing non-radiative losses in CQD optoelectronic devices.

Charge recombination also occurs at heterogeneous interfaces between the CQD layer and adjacent transporting layers, across which practical CQD devices require efficient charge transfer. Lattice strain and dangling bonds at these heterointerfaces generate interfacial trap states that act as recombination centers, pin the quasi-Fermi levels and suppress carrier injection efficiency [116]. Back injection from neighboring layers into the CQD film under operating bias further introduces parasitic currents that degrade device performance.

### 3.4. Environmentally Induced Degradation

The long-term stability of CQD films is limited at the surface. A large surface-to-volume ratio, undercoordinated surface atoms and a dynamic ligand shell make the surface the first site of attack by oxygen, water and heat, and the resulting surface defects introduce in-gap states that act as nonradiative recombination centers. As a consequence, exposure to these stressors lowers the PLQY and carrier mobility of the film, reduces device efficiency, and shortens operational lifetime. In this section, we examine how these environmental factors degrade CQD films and the theoretical approaches that have been used to mitigate their effects.

During the synthesis process, oxygen and moisture act mainly through competitive adsorption at undercoordinated surface sites. DFT calculations on 4 nm PbS CQDs showed that molecular O_2_ adsorbed at ligand vacancies induces deep in-gap states, whereas a fully passivated surface retains a clean band gap [117]. In PbS-EDT films, the DFT binding energy of H_2_O (0.48 eV) exceeds that of O_2_ (0.41 eV), which corresponds to an approximately 16-fold higher population of moisture-bound sites at room temperature, while oxygen displaces adsorbed moisture and p-dopes the hole transport layer [118]. QDSC fabricated in air at about 60 to 65% relative humidity (at 22 °C) showed PCE as low as about 5%, compared with about 10% below 25% relative humidity. At the scale of neighboring dots, molecular dynamics simulations of two hydroxylated PbS CQDs separated by 1 nm at 300 K indicate that hydrogen-bonded water bridges the dots and promotes epitaxial fusion, which offers a microscopic origin for the interband traps, larger Stokes shift and shorter carrier lifetime observed in films processed in humid air [119]. Thus, rational mitigation requires two complementary design rules, namely maximizing ligand coverage to block oxygen-induced deep states, and controlling humidity during processing [117,119]. For example, dry-air storage or meniscus-guided coating that expels ambient water to suppress water-mediated fusion and the resulting trap formation.

Heat degrades the film by weakening the ligand shell. Ligand binding is a temperature-dependent equilibrium with Gibbs energy. Zito and Infante decomposed the binding free energy into a ligand deformation term and an interaction term, with a coverage- and solvent-dependent solvation contribution [87], which makes clear that desorption begins at the weakest sites and that ligand choice should therefore be guided by site-resolved binding energies. For InAs CQDs, desorption energies of Cl^−^, Br^−^ and a 1:1 mixture were evaluated separately on facets, edges and vertices and guided a mixed-halide passivation. After 28 days in air, X-ray photoelectron spectroscopy showed 24.4% In_x_O_y_ and 23.8% As_x_O_y_ for the mixed-halide dots, against 42.6% and 43.1% for Cl-passivated dots, and arsenic oxidation resistance improved 2.9-fold relative to Br-passivated dots, with QDPD reaching an EQE of 75% at 1140 nm under 0.5 V bias [120]. A related approach showed by DFT that acetate binds to In-rich InAs (111) about 0.6 eV more strongly than bromide and reduces in-gap states. Adding methylammonium acetate as a final X-type treatment reduced the Stokes shift by 25%, nearly doubled the photoluminescence lifetime (4.83 to 9.36 ns), improved ink stability 4.4-fold under nitrogen, and gave a peak EQE of 37% at 950 nm [121].

The chemical space of candidate ligands is too large for case-by-case calculations, motivating the development of descriptor-based and machine-learning (ML)-assisted screening approaches. Zito and Infante described ligand selection as two coupled equilibria, binding at the surface and precipitation of the nanocrystal-ligand complex, each governed by a free energy that depends on surface coverage [87]. Ligands that maximize coverage can promote interdigitation and lower solubility, whereas highly entropic ligands stabilize the dispersion but may detach, so optimal ligands must balance both. Evaluating these free-energy cycles for every candidate is computationally demanding, so ML offers a practical shortcut: once trained on a sufficiently large set of computed ligand properties, it can predict suitable candidates within seconds and restrict experiments to the most promising ones [87]. By pairing automated high-throughput calculations with ML, ligand design can shift from empirical search to in silico prediction, which is particularly valuable for stability, where ligand choice determines how strongly the surface resists oxygen, moisture and heat.

## 4. Theoretical Models of CQD-Based Optoelectronic Devices

Although QDSCs, QDPDs and QLEDs stem from the same physical basis rooted in the quantum confinement of CQD materials, including size-tunable absorption or emission spectra, carrier hopping transport and recombination pathways, they adopt different design strategies depending on the target device function for achieving optimal performance. For QDSCs, design strategies focus on maximizing photon absorption and subsequent extraction of photogenerated carriers, while minimizing carrier recombination. On the other hand, QDPDs are designed to maximize selective absorption at target wavelengths and achieve fast extraction with minimal trap-assisted noise, as reverse-bias operation ensures nearly perfect charge extraction (~100%). Moreover, the introduction of charge multiplication within the CQD region surpasses the theoretical performance limit, enabling single-photon detection.

The design approaches in QLEDs promote radiative recombination by balancing charge injection and reducing carrier lifetimes, along with suppressing the non-radiative recombination that would otherwise quench emission. In this sense, various numerical simulation platforms have been developed to model CQD optoelectronic devices, each tailored to their respective device architectures and material properties. Solar cell capacitance simulator (SCAPS-1D) has been widely adopted for designing QDSCs owing to its computational efficiency in modeling multilayer structures under steady state and quasi-steady state. Meanwhile, Silvaco technology computer-aided design (TCAD) has become a standard tool for calculating all CQD optoelectronic devices because it enables multidimensional simulations incorporating charge transport, recombination, generation and spatial electric field distributions in complex device architectures. COMSOL Multiphysics offers a finite element-based framework that simultaneously couples optical, electrical and thermal phenomena, making it particularly suited for devices with irregular geometries or multiphysical interactions that are difficult to capture within the drift-diffusion systems of SCAPS and TCAD. These three platforms represent the most widely adopted tools for optoelectronic device simulations. However, additional simulation platforms and customized modeling approaches are also employed depending on the specific objectives, material systems and device architectures investigated in each study.

Although these platforms differ in their numerical implementation and supported physical models, they are rooted in the drift-diffusion transport framework, in which carrier transport is described by the coupled Poisson and carrier continuity equations given below.

Poisson’s equation:

$$\frac{d^{2}\phi \left(x\right)}{dx^{2}}=-\frac{q}{\varepsilon_{0}\varepsilon_{r}}(p-n+N_{D}^{+}-N_{A}^{-})$$

where *Φ*(*x*) is the electrostatic potential, *q* is the elementary charge, *ɛ*_0_ and *ɛ_r_* are the free space and relative permittivity, *p* and *n* are the hole and electron concentrations, $N_{D}^{+}$ and $N_{A}^{-}$ are the ionized donor and acceptor densities.

Continuity equations for electrons and holes:

$$\frac{1}{q}\frac{dj_{n}}{dx}=R_{n}-G_{n}$$

$$\frac{1}{q}\frac{dj_{p}}{dx}=G_{p}-R_{p}$$

where *j_n_* and *j_p_* are the electron and hole current densities, *G_n_* and *G_p_* are the generation rates for electrons and holes and *R_n_* and *R_p_* are the recombination rates for electrons and holes, respectively.

SCAPS-1D employs a 1D drift-diffusion framework coupled with a simplified optical model, enabling computationally efficient analysis of band alignment, defect density and interface recombination. The Poisson and continuity equations are solved utilizing material parameters including dielectric permittivity, doping concentration, electron affinity, bandgap, effective density of states, carrier mobilities and diffusion coefficients together with SRH lifetimes, trap characteristics, optical generation rates and radiative and Auger recombination coefficients. Full TCAD simulations such as Sentaurus and Atlas extend the same Poisson and continuity equations to 2D and 3D, providing finer control over trap physics and mesh geometry while enabling coupled process, electrical, thermal and optical simulations within a unified environment. This increased flexibility comes at the expense of substantially higher computational costs and greater setup complexity. However, accurate optical generation profiles in structures with strong interference or scattering typically require dedicated optical solvers beyond the conventional electrical TCAD framework. For planar multilayer structures, the transfer matrix method (TMM) provides a standard approach by solving Maxwell’s equations. It accounts for the propagation of electromagnetic fields across individual layers and boundary conditions at their interfaces, enabling efficient calculation of reflection, transmission and absorption spectra as well as spatially resolved optical generation profiles. These generation profiles are then incorporated into the continuity equations as source terms for electrical transport simulation. By discretizing Maxwell’s equations in space and time, finite-difference time-domain (FDTD) captures complex electromagnetic interactions and provides spatially resolved optical generation profiles for subsequent electrical simulations. It is particularly useful when the planar assumption of TMM breaks down, particularly in nanostructured, textured and plasmonic geometries where scattering, diffraction and near-field effects become significant, although this flexibility comes at substantially higher computational cost.

The material parameters required by the device-level platforms described above are obtained through a combination of atomistic and experimental inputs. DFT-level calculations provide intrinsic quantities including bandgap, electron affinity, effective density of states and dielectric permittivity, which set the electrostatic boundary conditions in Poisson’s equation and the optical constants used in TMM/FDTD-derived generation profiles. Transport- and defect-related parameters (carrier mobility, SRH lifetimes, trap densities, interface recombination velocities), however, are governed by disorder, ligand chemistry and inter-particle coupling effects not captured at the single-particle DFT level and are instead calibrated against experimental measurements (e.g., mobility from Hall/FET measurements, trap dynamics from transient photoluminescence or admittance spectroscopy) before being used as inputs to device-level simulation tools such as SCAPS-1D and TCAD. Because both DFT-derived and experimentally calibrated parameters carry uncertainty, this uncertainty propagates to the device level and is assessed through parametric sensitivity sweeps over physically justified parameter ranges rather than a single fixed value set.

### 4.1. CQD-Based Solar Cells

#### 4.1.1. Working Principle

The operating principle of QDSCs begins with the absorption of incident photons with sufficient energy higher than the CQD bandgap, which excites electrons from the VBM to the CBM and thereby creates electron–hole pairs within the CQDs. These electron–hole pairs are spatially separated when they encounter the energy offsets at the CQD/CQD and CQD/transporting layer interfaces and subsequently transported to their respective contacts to produce an external photocurrent.

The power conversion efficiency (PCE), the primary performance metric for QDSCs, depends jointly on three factors: the open-circuit voltage (*V_OC_*), determined by non-radiative recombination losses that narrow the quasi Fermi level splitting; the fill factor (*FF*), constrained by resistive and recombination losses under operating conditions; and the short-circuit current density (*J*_SC_), set by the efficiency of carrier generation and extraction [92,122]. The theoretical upper bound on the PCE of QDSCs is assessed through detailed balance analysis, which forms the basis of the Shockley-Queisser limit and predicts a maximum PCE exceeding 30% for a single junction with a 1.1 eV bandgap [123].

The first demonstration in a QDSC, reported in 1998, employed a sensitized architecture consisting of a CQD layer, serving as the light absorber, deposited onto a nanoporous TiO_2_ scaffold that functioned as the electron acceptor [124]. A multilayer CQD-sensitized QDSC proposed by Im et al. incorporates deposited HgTe CQDs with partial coverage of the TiO_2_ surface (Figure 4a) [125]. Because CQDs absorbed onto the TiO_2_ surface achieved a submonolayer coverage, poor efficiency of photon absorption restricted the PCE, motivating the formation of a thick CQD absorbing layer. This led to the development of the Schottky junction QDSC [126], in which a CQD film was directly sandwiched between two electrodes, with charge separation driven by the depletion region at CQD/electrode interfaces (Figure 4b). Unfortunately, the high density of trap states at the CQD/electrode interfaces causes Fermi-level pinning, limiting the built-in potential regardless of the electrode’s work function and thereby reducing the achievable *V_OC_* [81]. Moreover, because the depletion region is confined to the Schottky interface, charge carriers generated away from the junction rely primarily on diffusion for extraction, thereby increasing the recombination rates. The depleted heterojunction QDSCs have been suggested to establish a wider depletion region, improving charge separation and extraction, while increasing the built-in potential. Energy level engineering ensures the specific modulation of band alignment and carrier distributions, facilitating carrier transport in depleted heterojunction QDSCs. Moreover, the introduction of a buffer layer at CQD/transporting layer interfaces improves the photovoltaic performance by suppressing charge recombination and improving *V_OC_* (Figure 4c). Moreover, the introduction of bulk heterojunctions, which create an interpenetrating network between n-type and p-type CQDs, forms an enlarged depletion region, enabling efficient charge extraction and suppressing charge recombination (Figure 4d). This strategy further improves the light harvesting capabilities as it allows the use of thicker CQD layers. Recent advances in QDSCs include multi-junction (tandem) structures proposed to surpass the Shockley-Queisser efficiency limit by stacking subcells with complementary bandgaps. This structure enables each subcell to absorb different regions of the solar spectrum, while recombination layers facilitate charge transfer between neighboring subcells, leading to improved solar energy harvesting. The tandem architecture combining an organic PTB7-Th: IEICO-4F front cell with a PbS CQD back cell successfully achieves an outstanding PCE of 13.7%, exceeding those of single-junction organic and CQD-based solar cells (Figure 4e).

The multiple exciton generation (MEG) technique proposes an innovative strategy for overcoming the Shockley-Queisser limits within a single-junction solar cell architecture [19,115]. In conventional bulk semiconductors, absorption of a photon generates only a single electron-hole pair, regardless of photon energy. This excess energy above the bandgap dissipates as heat during the relaxation of photogenerated carriers to the band edges, rather than contributing to the generation of additional free electrons. Meanwhile, discrete energy levels of CQDs allow the excess energy to be utilized for the generation of free electrons, thereby mitigating thermalization loss and creating multiple electron-hole pairs. The demonstrations of MEG in CQDs [129,130] have highlighted its potential to increase the theoretical efficiency limit of single junction structures to approximately 44% [131].

#### 4.1.2. Simulation Modeling of QDSCs

Simulation studies of QDSCs have mainly focused on optimizing performance to approach the Shockley-Queisser efficiency limit by tuning architecture parameters such as layer thickness, doping concentration and bandgap (Table 1).

##### PbS CQD-Based QDSCs

The largest cluster of simulation work in the reviewed literature targets PbS CQD absorbers, reflecting their tunable bandgap, high absorption coefficient and relatively mature ligand-exchange processing [94,147]. Pandey et al. calibrated a SCAPS-1D model of the Au/PbS CQD-EDT/PbS CQD-TBAI/ETL/ITO stack directly against an experimentally realized device and demonstrated that replacing a conventional TiO_2_ electron transporting layer (ETL) with Mg-doped ZnO (MZO) raises the simulated PCE to 15% [144]. Building on this baseline, Khanna et al. focused on a wide-bandgap (E_g_ = 1.56 eV) PbS-CQD absorber and mapped the design space defined by CQD thickness and bulk defect density, showing that PCE is far more sensitive to bulk defect density than to CQD thickness once the latter exceeds the optical absorption depth, with a representative PCE of 13.14% reported [141]. Salunke et al. developed simulation models that couple SCAPS-1D with TMM to systematically investigate the design principles in QDSCs [137]. This work established how the energy landscape governs device performance by revealing the interplay among bandgap combinations, electron affinity offsets (Δ*E_a_*), carrier mobility and optical absorption. These simulations elucidated that superior performance depends not only on maximizing spectral coverage but also on maintaining favorable energy alignment, as excessively large bandgap differences hinder charge extraction. Instead, an optimized PbS CQD ensemble achieved the best balance between broadband light absorption and efficient carrier extraction. Furthermore, this study demonstrated that high carrier mobility in CQD solids is essential for approaching the theoretical performance limit. Based on these simulation-guided optimizations, they predicted a maximum PCE of 16.48% under AM 1.5 G illumination, providing valuable guidelines for designing multi-bandgap QDSC.

A second generation of PbS CQD studies focused on transporting layer engineering rather than the CQD absorber itself. Kumar et al. compared conventional (ITO/TiO_2_/PbS CQD-TBAI/PbS CQD-EDT/HTL/Au) and inverted (ITO/TiO_2_/PbS-TBAI/HTL/Au) architectures [140]. Several hole-transporting layer (HTL) candidates, including CuI, MoS_2_, MoO_3_, CuO, Spiro-OMeTAD and PbS-EDT, have been explored to identify optimal QDSC structures. Their simulations showed that eliminating the PbS-EDT hole-selective interlayer and directly interfacing the PbS-TBAI absorber with an optimized HTL, particularly MoO_3_, reduced interfacial recombination and improved carrier extraction. As a result, the optimized inverted device achieved a PCE of 16.4%, although this remained lower than the best-performing MZO-based design discussed previously. Pandey et al. evaluated the buffer layer inserted between the FTO front contact and the TiO_2_ ETL, screening ZnS, Ag_2_S, CdS and ZnSe [135]. They suggest that a higher buffer bandgap improved the *V_OC_*, with the ZnS buffer layer achieving the highest PCE of 17.1% due to a reduced conduction band offset at the buffer/TiO_2_ interfaces. Naureen et al. directly compared WO_3_ and WS_2_ as ETL alternatives in a Sb_2_Se_3_/PbS/CdS-buffer structure [139]. As a result, this study shows that WS_2_ outperformed WO_3_ as an ETL, achieving an optimized PCE of 20.6%. Kim et al. combined TMM optical simulations with SCAPS-1D to investigate the influence of optical interference on QDSCs [146]. Their simulations revealed that Fresnel reflection and standing-wave interference between the ZnO ETL and PbS CQD absorber significantly influence the light absorption profile, causing photocurrent saturation and leading to an underestimation of the optimal CQD thickness. By introducing a textured ZnO layer to suppress interference, they demonstrated that optical optimization alone increased the optimal CQD thickness, enhanced *J*_SC_ and improved the PCE from 9.9% to 11.1%. Kim et al. [143] and Lee et al. [138] both employed the SCAPS-1D simulator to investigate hybrid CQD/organic solar cells with different interfacial ligand treatments at the ZnO/CQD interface. Their simulations demonstrated that ligand engineering effectively modulates interfacial potential barriers, thereby improving charge transport and reducing recombination losses, consistent with experimental validation of the outstanding PCEs of 13.74% and 14.1%, respectively. Hasnain et al. reported the highest PCE of 32.9% on a Cu_2_O/PbS CQD/Ag_2_S/FTO structure using the SCAPS-1D solver [134]. This study systematically compared PbS CQDs with PbSe CQDs, identifying distinct optimum doping concentrations and reporting PCE values of 28% and 26.2%, respectively, while the cascade Ag_2_S/PbS/Cu_2_O configuration further increased the PCE to 32.9%. This result represents the highest simulated performance among the reported studies. These values should be regarded as theoretical upper bounds rather than experimentally demonstrated efficiencies, as the simulations are limited to the idealized transport equations of SCAPS-1D and exclude additional resistive and stability characteristics.

##### Pb-Free CQD-Based QDSCs

Eco-friendly QDSCs have emerged as alternatives to address the toxicity and commercialization challenges. Although numerous perovskite-based QDSCs have been proposed and demonstrated, relatively few studies have focused on CQDs in recent years. Recently, Park et al. developed Pb-free QDSCs by introducing a p-type InAs CQD layer beneath the n-type InAs CQD absorbing layer to form an all-InAs homojunction [136]. They established a simulation framework that combines DFT calculations and TMM simulations to guide ligand engineering and device design. The DFT calculations revealed that ZnCl_2_ ligands suppress the formation of donor defects, thereby lowering the level of self-doping, while TMM simulations identified the optimal CQD thickness that maximizes light absorption and demonstrated that the p-type InAs CQD layer acts as an optical spacer to enhance infrared absorption. Despite achieving a modest PCE of 2%, this work demonstrated the potential of computationally assisted design for emerging Pb-free CQD photovoltaics. Using SCAPS-1D, Hakami investigated a lead-free QDSC based on AgBiS_2_-ZnS-ZnSe dual-shell quantum dots in an ITO/SnO_2_/BaTiO_3_(polarized)/AgBiS_2_-ZnS-ZnSe/Li: NiO_x_/Au architecture, in which an ultra-thin (4 nm) polarized BaTiO_3_ interlayer was inserted between the ETL and absorber to generate a polarization-induced internal electric field that enhances charge separation and suppresses interfacial recombination [133]. Comprehensive optimization of absorber thickness, acceptor concentration, defect density and transport-layer properties yielded a PCE of 28.43%, illustrating that dielectric interface engineering can be a powerful complementary lever to absorber-layer optimization in Pb-free CQD solar cells. Separately, SCAPS-1D has also been used to directly compare bulk and quantum-confined absorber configurations within the same Pb-free material system: Mehrabian et al. modeled Ag_2_S solar cells across a size range spanning bulk, 10 nm, 5 nm, 3 nm and 2 nm QD absorbers, incorporating size-dependent band gap, electron affinity and effective density-of-states inputs to isolate genuine quantum-confinement effects from other device parameters [132]. Bulk Ag_2_S and 10 nm QDs achieved the highest simulated efficiencies (16.60% and 16.86%, respectively), while smaller QDs exhibited higher built-in potentials and *V_OC_* due to stronger confinement but suffered from intensified recombination losses and optimal absorber thickness decreased sharply with QD size. The authors themselves caution that, because SCAPS-1D treats the sub-10 nm QD absorber as a homogeneous effective medium and cannot explicitly capture ligand chemistry, inter-dot tunneling, or energetic disorder, these smaller-QD results should be interpreted as theoretical sensitivity analyses of confinement trends rather than quantitative predictions for fabricated devices.

##### Tandem QDSC Architectures

The spectral losses inherent to single-junction QDSCs, including thermalization and transmission losses, motivate tandem architectures in which CQDs serve as subcells to extend spectral utilization and improve overall device efficiency. Kashyap et al. reported the first demonstration of a two-terminal a-Si: H/PbS CQD monolithic tandem structure using TCAD, combining a wide-bandgap (1.7 eV) a-Si: H top cell with a narrow bandgap (1.2 eV) PbS CQD bottom cell. The optimization of the absorbing layer thicknesses to 200 nm (top) and 150 nm (bottom) for current matching yielded a *J*_SC_ of 10.52 mA·cm^−2^, a *V_OC_* = 1.59 V, an *FF* = 71.65% and a PCE = 12.0% [145]. Madan et al. extended this tandem architecture to a perovskite/PbS-CQD pairing by optimizing the perovskite top cell under standalone conditions and using its filtered transmission spectrum to simulate the PbS-CQD bottom cell [142]. This tandem architecture achieved a simulated PCE of 23.36%, highlighting the potential of perovskite/PbS CQD integration for low-cost tandem photovoltaics.

Two additional TCAD studies examined non-CQD tandem and hybrid architectures that were included in the reviewed set as methodological benchmarks for CQD-based tandem device modeling using Silvaco. Bouhdjar et al. simulated a micromorph a-Si:H/µc-Si:H tandem in both two-terminal and four-terminal configurations, reporting initial and degraded efficiencies that highlight the importance of incorporating stability considerations into tandem device simulation [148]. Additionally, an air-processed ZnO nanorod/CH_3_NH_3_PbI_3_/spiro-OMeTAD perovskite cell provided an experimental benchmark, with simulated performance validated against a fabricated device (PCE ≈ 10.2%) and HTL doping linked to enhanced charge transport [149].

Recent QDSC studies predominantly employ SCAPS-1D, whereas TCAD remains relevant for transient, noise and non-PV response analyses, including QDPDs and the strain- and band-structure-limited III-V epitaxial QD-on-Si case discussed earlier. The combination of SCAPS-1D and TMM further provides a practical means of integrating electrical and optical modeling, enabling more accurate assessment of light absorption, interference effects and overall photovoltaic performance.

### 4.2. CQD-Based Photodetectors

#### 4.2.1. Working Principle

QDPDs share the same fundamental photophysical processes as QDSCs, including light absorption, photocarrier generation, separation, transport and extraction. However, their distinct operating objectives demand different device architectures and optimization strategies. QDSCs are designed to maximize conversion efficiency from light power to electrical power, whereas QDPDs prioritize high sensitivity capable of detecting small optical signals.

QDPDs are broadly classified into photoconductors, phototransistors and photodiodes according to their device architectures and carrier collection mechanisms [150,151]. Photoconductors represent the simplest device architecture, in which a CQD solid is directly contacted by two electrodes and photogenerated carriers are collected under the applied electric fields. This architecture gives rise to photoconductive gain when one type of photogenerated carrier remains trapped for a prolonged period. These long-lived trapped carriers ensure that the mobile carriers traverse the device multiple times before recombination, enabling EQE exceeding 100% [152,153]. However, an increase in trapped carriers inevitably facilitates trapping-detrapping processes, elevating noise current and reducing operational speed [152,154,155,156].

Phototransistors incorporate a gate electrode to electrostatically modulate carrier transport in the CQD channel [157]. Under illumination, photogenerated carriers tend to accumulate in the local CQD region, thereby modulating the channel conductivity. An increase in channel conductivity amplifies the source-drain current through the photogating effect, enabling ultrahigh responsivity under weak light conditions [158]. However, the slow dissipation of accumulated carriers limits the response speed, leading to poor detectivity.

Alternatively, photodiodes employ a rectifying junction to form an internal electric field for efficient carrier separation and extraction, while suppressing the capture of photogenerated carriers by defect states. This rectifying structure ensures low noise current, fast response speed and compatibility with CMOS readout circuits [159,160,161,162]. However, the absence of charge multiplication limits the maximum EQE to 100%, as each absorbed photon ideally generates only a single electron-hole pair, making the photodiodes unsuitable for weak-light detection [152].

Various carrier multiplication strategies have been explored to achieve ultrahigh performance, enabling single-photon-scale detection (SPD) in QDPDs. The MEG mechanism, discussed in Section 4.1.1, relies on the generation of multiple electron-hole pairs from the absorption of a single photon with high energy. This mechanism has been experimentally demonstrated in PbSe and PbS CQDs with quantum yields exceeding 300% [163], indicating that a single absorbed photon generates more than three electron-hole pairs. However, the practical implementation of MEG in QDPDs remains challenging because the requirement of high-energy photons above the MEG threshold restricts the multiplication gain and hinders its application in the infrared region.

Recently, impact ionization has emerged as a viable route for charge multiplication in QDPDs because it relies on carrier acceleration under the high electric field rather than the excitation of high-energy photons [18,164]. Avalanche photodiodes (APDs) typically employ p-n or p-i-n structures to form a wide depletion region with a strong electric field, where photogenerated electrons are efficiently accelerated. In CQD-based APDs, ligand engineering provides an effective approach to control the doping concentration of CQD solids, enabling the formation of well-defined p-n junctions. The first demonstration of CQD-based APDs achieved a multiplication gain exceeding 85 through the optimization of the CQD thickness and dot-to-dot distances [40], which facilitated efficient electron hopping and avalanche multiplication. Further integration of ligand engineering and CQD size control into separated absorption and charge multiplication (SACM) architectures provides a viable pathway toward realizing single-photon avalanche photodiodes (SPADs) [165,166].

QDPD performance is commonly benchmarked using internal gain, noise current, responsivity and specific detectivity. Internal gain (*G*) describes the number of carriers collected per absorbed photon, arising from photoconductive trapping, phototransistor photogating, or avalanche/impact-ionization multiplication. In devices where avalanche multiplication is the dominant or sole gain mechanism, *G* is often denoted specifically as the avalanche (multiplication) gain, *M*, to reflect its definition within impact-ionization theory. It is different from responsivity (*R*), the ratio of the photocurrent to the optical power, given below:

$$R=\frac{I_{ph}}{P_{opt}}=\frac{\eta q}{h\nu }\;(A/W)$$

where *I_ph_* is the photocurrent, *P_opt_* is the optical power, *η* (or EQE) is the quantum efficiency defined by the number of electron-hole pairs generated per incident photon, *h* is Planck’s constant and *υ* is the photon frequency. For a given EQE, the responsivity increases linearly with wavelength. It indicates how effectively a detector converts incoming light into an electrical signal [41]. Specific detectivity (D*) measures a photodetector’s ability to sense weak optical signals in the presence of noise and since noise directly determines D*, its physical origins must be established first.

In a photodetector in general, the noise current that ultimately limits detectable signal is not a single quantity but the sum of several physically distinct contributions, including thermal noise (or Johnson noise), shot noise, flicker noise and multiplication noise. In particular, thermal noise *S_th_* = 4*k_B_T*/*R_s_* arises from thermally induced carrier motion in resistive elements (with device resistance *R_s_*), which is bias-independent and exhibits a flat, wide-bandwidth power spectral density [41]. Shot noise *S_shot_* = 2*qI* originates from the discrete nature of the charges carried by electrons and holes. With no bias applied, the device current (*I*) is zero and the spectrum closely matches the thermal noise spectral density. Flicker noise (or 1/*f* noise), in another hand, is commonly attributed to carrier number fluctuations from trapping-detrapping events at defect states with a broad distribution of time constants and dominates at low frequency [167]. CQD films are especially susceptible to flicker noise because of their high surface-to-volume ratio, ligand-shell trap states spanning a wide range of binding energies, single-dot charge blinking and dot-to-dot energetic disorder from size polydispersity, all of which broaden the effective trap-time distribution. Because thermal and shot noise remain frequency-flat while flicker noise decays as 1/*f*, the two regimes intersect at a corner frequency *f_c_*, below which flicker noise dominates and D* degrades toward low frequency and above which the device enters the more predictable white-noise-limited regime that is a primary reason high-speed operation is generally favored for CQD photodetectors [168,169]. In devices employing avalanche multiplication, the responsivity gain from carrier multiplication is accompanied by an excess noise factor (*F*), which quantifies the additional noise arising from the statistical spread in the number of impact-ionization events each carrier undergoes before exiting the multiplication region. *F* generally increases with avalanche gain *M*, following *F*(*M*) ≈ *kM +* (2 − 1*/M*)(1 − *k*) where *k* is the ionization coefficient ratio between holes and electrons. Specific detectivity is defined from the noise-equivalent power (NEP) normalized by detector area (*A*) and electrical bandwidth (*B*), given by:

$$D^{*}=\frac{\sqrt{AB}}{NEP}=\frac{R\sqrt{AB}}{i_{n}}(Jones)$$

where *NEP* = *i_n_*/*R* with *i_n_* is the total noise current spectral density summing all of the contributions above. An increase in gain and responsivity improves D* only if the accompanying rise in noise does not grow proportionally or faster. In avalanche devices, *F* increases with *G*, so pushing gain higher can raise *i_n_* enough to leave D* unchanged or even reduced. Therefore, especially in SPD, high gain is not sufficient evidence of SPD capability, but one must consider low excess multiplication and flicker noise.

Depending on how the noise is determined, there are generally two approaches for obtaining D*. First, if noise includes direct experimental measurement of the noise spectral density, then D* is calculated via the above formula. Alternatively, under the assumption of a shot-noise-limited condition, *i_n_* is attributed solely to shot noise, such as *i_n_* = *S_shot_* = 2*qI*. In this case, D* can be calculated using theoretical *R* and EQE. This shot-noise-limited formulation is convenient because it requires no dedicated noise measurement and can be evaluated directly from device simulation outputs (e.g., TCAD- or SCAPS-1D-derived dark current and EQE), but it represents an upper-bound estimate of D* rather than a measured quantity, since it implicitly assumes shot noise is the sole or dominant noise source and therefore omits flicker and multiplication noise that are often significant in CQD films that can substantially lower the true detectivity of CQD-based devices [168]. We will annotate Table 2 to distinguish detectivities obtained from measured noise spectra from those estimated using shot-noise-limited models.

#### 4.2.2. Simulation Modeling of QDPDs

##### Charge-Multipliable QDPDs

Because charge transport in CQD films is strongly influenced by electric fields and trap states, designing internal gain mechanisms such as avalanche multiplication and photomultiplication (PM) has become one of the most simulation-intensive research areas (Table 2).

Yang et al. provided a comprehensive demonstration of the SACM PbS/ZnO APD, combining TCAD simulation with experimental validation [166]. These simulations show that, without an intentional charge layer, the electric field extends into the CQD absorption region and increases band-to-band tunneling leakage; introducing a p-type CQD charge layer confines the field within the intrinsic ZnO multiplication layer, allowing it to exceed the critical impact ionization field and facilitating avalanche gain while suppressing tunneling. This field engineering rule yields a gain-normalized dark current density of 4.86 × 10^−7^ A cm^−2^, reported as the lowest among CQD photodetectors with internal gain, together with a D* of 1.15 × 10^12^ Jones and an avalanche gain of 2.3, illustrating that higher gain does not necessarily translate into higher detectivity once excess multiplication noise is taken into account. Kim et al. advanced this approach by demonstrating kinetically pumped avalanche multiplication, supported by complementary TCAD, DFT and COMSOL simulations [40]. This represents the first demonstration of avalanche multiplication within the CQD region rather than relying on MEG. By systematically tuning the dot-to-dot distance through the modifying ligand species, they balance impact ionization against electron hopping in PbS CQD layers, achieving a maximum multiplication gain of 85, a peak detectivity of 1.4 × 10^14^ Jones, a bandwidth of 1.1 MHz and a linear dynamic range of 124 dB. Additional simulation findings indicate that a CQD layer thickness exceeding 540 nm is required to sustain efficient charge multiplication while suppressing noise current, providing a design guideline for CQD-based APDs.

Bai et al. demonstrated a complementary low-voltage approach to gain enhancement based on photon multiplication (PM) through enhanced tunneling injection using a thin (2–6 nm) Au nanoparticle (NP) interlayer between two PbS CQD layers (ITO/ZnO/PbS/Au-NPs/PbS/P3HT/Ag), with COMSOL simulations used to optimize the electric-field and absorption distribution around the nanoparticles [172]. The Au NPs simultaneously enhance near-field absorption through localized surface plasmon resonance and serve as controllable hole traps that promote electron tunneling from the Ag electrode, achieving a responsivity of 1088.86 A·W^−1^, an EQE of 138,006% (218-fold higher than the non-plasmonic control devices) and a detectivity of 1.2 × 10^14^ Jones at only −1 V, compared with the −50 to −60 V typically required by earlier PM type QDPDs [178,179].

An innovative approach couples a CQD photoconversion layer with an amorphous selenium (a-Se) HTL supporting hole impact ionization, with TCAD simulations comparing CdSe/a-Se architectures with and without a CeO_2_ interlayer [34]. The optimized hybrid device reaches a specific detectivity ranging from 0.5 to 5 × 10^12^ Jones, a fast 3-dB bandwidth of 22 MHz, a low dark current density ranging from 5 to 10 pA·cm^−2^ and a wide linear dynamic range of 130~150 dB. This result elucidates that impact ionization gain was engineered in an adjacent wide bandgap chalcogenide layer rather than in the CQD absorbing layer itself.

Another study used SCAPS-1D to optimize the device structure for reduced dark current density by sweeping the bandgap of the PbS-EDT (1,2-ethanedithiol-treated) HTL in an ITO/ZnO/PbS-halide/PbS-EDT/Au photodiode, finding an optimal E_g_ of 1.4 eV that produced a favorable gradient band structure with minimized dark current density. The fabricated device based on this optimized design achieves a dark current density of 265 nA·cm^−2^, a responsivity of 0.65 A·W^−1^, a detectivity of 5.73 × 10^12^ Jones and an EQE of 66.5% at 1300 nm, confirming SCAPS-1D as an effective low-cost design tool even though it was originally developed for solar cells. Parmar et al. further developed a transient rate equation model that directly reproduces the time-resolved photocurrent of a p-i-n PbS photodiode as a function of the ligand concentration during the fabrication processes [175]. This study provides the first reported quantitative agreement between transient Setfos simulations and experimental photocurrent measurements in QDPDs, revealing a minimum dark current at an intermediate ligand concentration and providing a direct simulation-derived guideline for QDPD fabrication.

##### Hybrid Heterojunction QDPDs

**Figure 5:**
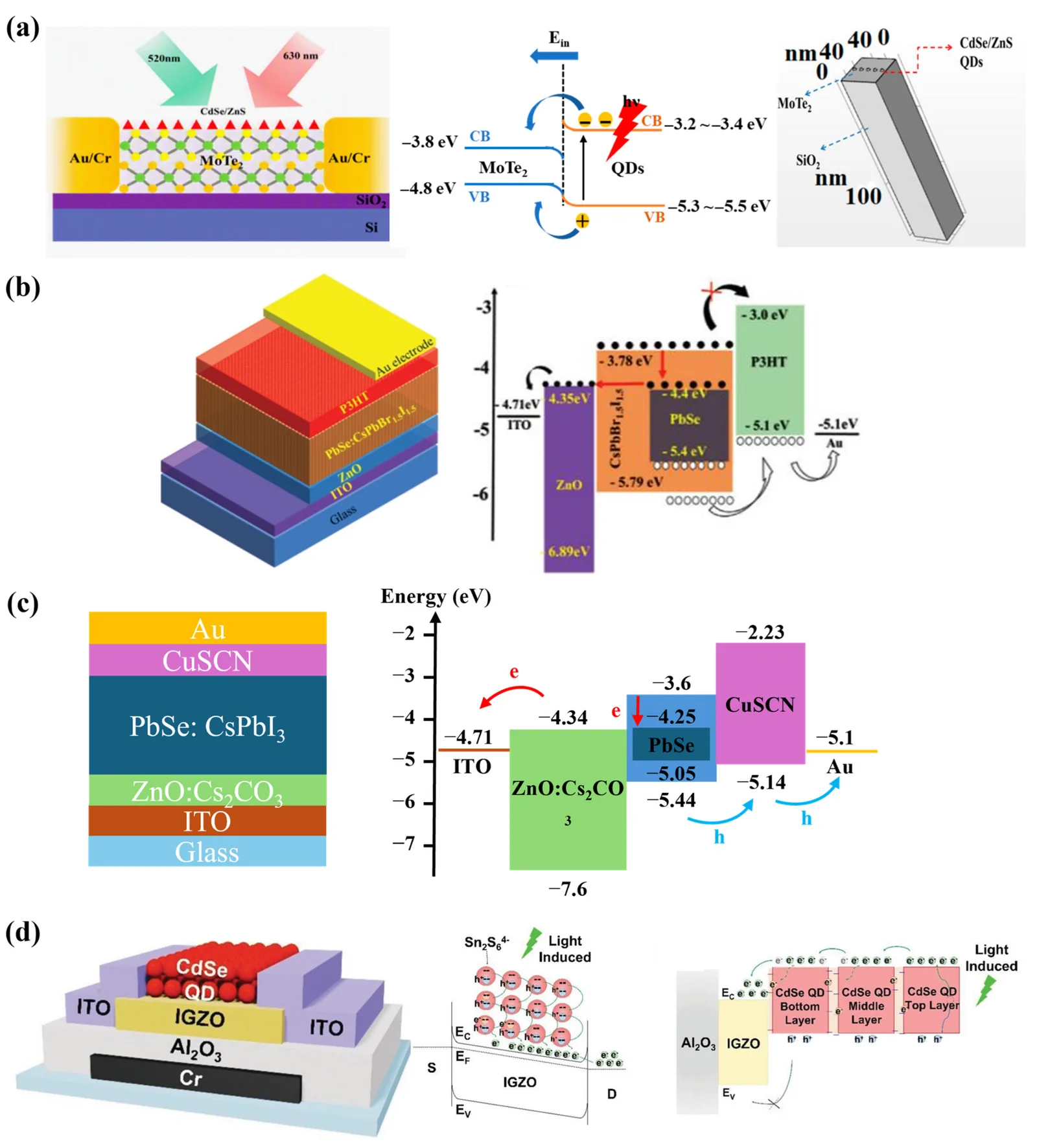
Illustration of device architecture, energy band of (**a**) CdSe/ZnS/MoTe_2_ hybrid QDPD (reproduced from Ref. [170] under the terms of the Creative Commons Attribution 4.0 (CC BY 4.0) license), (**b**) ITO/ZnO/PbSe CQD: CsPbBr_1.5_I_1.5_/P3HT/Au hybrid bulk-heterojunction QDPD (reproduced with permission [177]. Copyright 2022 John Wiley and Sons), (**c**) ITO/ZnO: Cs_2_CO_3_/CsPbI_3_:PbSe CQD/CuSCN/Au QDPD [174] and (**d**) CdSe CQD-based hybrid phototransistor (reproduced with permission [176]. Copyright 2022 John Wiley and Sons).

Simulation tools are also used for examining interfacial charge transfer mechanisms in complex heterostructures. Hussain et al. combined COMSOL, TCAD and DFT simulations to model a CdSe/ZnS sensitized MoTe_2_ hybrid photodetector [170]. COMSOL simulations revealed that broadband enhancement of visible absorption originated from CQD exciton absorption superposed with Fabry–Pérot interference in the underlying SiO_2_ layer, while DFT and TCAD confirmed a staggered type-I band alignment at the CdSe/ZnS/MoTe_2_ interface that promotes photogating-driven photocurrent enhancement (Figure 5a). Despite this multiscale rigor, the reported EQE (0.31~0.39%) and responsivity (1.3–2.0 mA W^−1^) remain low compared with the other device classes in this review. This limitation arises from the intrinsically weak optical absorption of thin MoTe_2_ channels and carrier losses associated with defect- and trap-mediated recombination in MoTe_2_ and CQD/2D interfaces [180].

By contrast, the Sulaman group demonstrated close agreement between TCAD simulations and experimental results in both a PbSe CQD-decorated CsPbI_3_ nanorod photodetector and an earlier PbSe CQD: CsPbBr_1.5_I_1.5_ bulk-heterojunction device, using the simulations to guide ETL engineering through ZnO/Cs_2_CO_3_ blending and dedicated carrier extracting layers, respectively [174,177]. A CsPbI_3_: PbSe device reaches a responsivity of 7.64 A·W^−1^ and a detectivity of 5.23 × 10^14^ Jones in self-powered mode, making it one of the highest self-powered detectivities in the entire dataset, while the PbSe: perovskite BHJ device achieves 6.16 A W^−1^ and 5.96 × 10^13^ Jones (Figure 5b,c).

Park et al. applied TCAD simulations to extract the interface density of states in a CdSe CQD/IGZO hybrid phototransistor by comparing Sn_2_S_6_^4−^ chalcometallate ligands with conventional SCN^−^ ligands (Figure 5d) [176]. These simulations reproduced the experimental electron concentration, band bending and electric field distribution, quantitatively confirming a lower interfacial trap density for the Sn_2_S_6_^4−^-capped device. This reduction in trap density translates into an exceptionally high responsivity of 2.12 × 10^4^ A·W^−1^ and detectivity of 2.74 × 10^16^ Jones, making it, to the best of our knowledge, the highest detectivity.

##### Multispectral and Multicolor QDPDs

A distinct engineering objective, wavelength-selective multicolor detection from a single pixel, has motivated a smaller number of studies that couple rate-equation and quantum transport solvers rather than conventional drift-diffusion TCAD simulations. A representative example designs a multi-color mid-infrared (MIR) QDPD using two differently sized InSb/ZnSe core/shell QDs to target the 3 µm and 4 µm bands simultaneously [173]. They self-consistently solve coupled rate equations together with a three-dimensional Schrödinger-Poisson model, cross-validating the results against a non-equilibrium Green’s function (NEGF) calculation and an experimentally fabricated reference device. The device exploits resonant tunneling through wavelength-selective quantum-well contacts deposited on interdigitated electrodes to suppress dark current, reporting specific detectivities of 9 × 10^10^ Jones and 1.5 × 10^11^ Jones for 4 µm and 3 µm, respectively. This represents the first QDPDs capable of simultaneously resolving two distinct MIR wavelengths through interband transitions.

Table 2 compares the performances of all simulation-based studies that have been discussed above. Our investigation suggests that charge multiplication architectures dominate the highest detectivity results. The avalanche [40,166] and PM [172] devices, together with the interface-optimized phototransistor [176], achieve the highest detectivities, indicating that internal gain engineering offers the greatest performance for improving QDPD performance when the underlying noise mechanisms are accurately modeled.

In recent years, TCAD simulation has emerged as an increasingly important tool for modeling and interpreting QDPD behavior. However, because commercial TCAD platforms lack native material models for the disordered, trap-dominated transport intrinsic to CQD solids, such as hopping conduction, multi-trap Poole-Frenkel behavior and quantum-confinement effects, TCAD is rarely used in isolation. Instead, it is increasingly combined with complementary simulation tools, such as DFT for accurate band structure and defect-level calculations, COMSOL and FDTD for optical field distribution, SCAPS-1D for rapid band alignment screening and Setfos for transient/rate-equation modeling, to build a more physically complete and self-consistent picture of device operation. This multi-tool approach compensates for TCAD’s inherent modeling gaps and enables a more accurate explanation of experimentally observed performance metrics.

### 4.3. CQD-Based Light-Emitting Diodes

#### 4.3.1. Working Principle

CQDs possess a unique combination of high PLQY, long photoluminescence lifetimes, large Stokes shifts, high chromaticity and narrow, size-tunable emission linewidths, making them attractive light-emitting materials for wide-color-gamut displays and general lighting [43]. These QLEDs exhibit two types of distinct operating principles: color-converting CQD layers (a photoluminescent configuration) and light-emissive CQD layers (an electroluminescent configuration). In the latter case, a light-emissive CQD layer (EML) is sandwiched between an electron injection layer (EIL) and a hole injection layer (HIL), together referred to as charge injection layers (CILs). Upon applying the electric field, electrons and holes are injected into the EML through the EIL and HIL, respectively, where they undergo radiative recombination, creating photons with energies determined by the CQD size and composition. The narrow emission linewidth enabled by the monodisperse CQD size distribution allows self-emissive QLEDs to reproduce color coordinates spanning almost the entire Rec. 2020 gamut, achieving a level of color purity that is difficult to attain with conventional phosphor or organic emitters [181].

QLED architectures are categorized based on the direction for initiating light emission, which determines the position of the transparent conductive electrodes [42]. ITO has been widely utilized for transparent conductive electrodes due to its superior optical transparency in the visible spectrum and excellent electrical conductivity. However, its sputtering process inevitably degrades the underlying organic layer when the ITO electrode is stacked on top of the organic layer. The bottom emission (BE) geometry has been adopted to position the ITO electrode adjacent to the glass substrate, allowing light to be emitted through the glass/ITO stack. Alternatively, top emission (TE) architecture emits light upward through a thin metal top electrode with sufficient semitransparency, relaxing the optical requirements on the substrate side. Furthermore, QLED architectures are also classified according to the polarity of the bottom electrode as either normal or inverted structures. Early BE-QLEDs employed ITO as a high work function anode to favor efficient hole injection, whereas the inverted architecture, which later became the standard configuration, places a solution-processed ZnO EIL beneath the QD film to improve the balance between electron and hole injection and enable the efficiency improvements discussed below [182,183].

Power efficiency is a key performance metric for QLEDs, representing the ratio of the emitted optical power to the electrical input power. The EQE, which directly contributes to power efficiency, is expressed as *η*_EQE_ = *η*_IQE_ × *η*_OCE_, where *η*_IQE_ and *η*_OCE_ denote the internal quantum efficiency (IQE) and the outcoupling efficiency (OCE), respectively [184]. Recent advances in QLEDs have enabled the IQE to approach 100%, leaving optical outcoupling as the principal remaining bottleneck [185,186,187]. For isotropic emitters, theoretically EQE is restricted to approximately 30% due to photon losses associated with optical outcoupling [188].

Operational lifetime, defined as the duration over which a device maintains its performance under continuous driving, is another critical performance metric, since a device combining high efficiency with rapid degradation offers limited practical utility for displays or lighting applications. Lifetime is typically evaluated by driving a device at a constant current density and monitoring the decay of luminance over time. Two parameters for lifetime are conventionally used: T_50_ and T_95_, defined as the time required for luminance to decay to 50% or 95% of its initial value, respectively [189]. Lifetime performance shows a strong dependence on emission color. Red and green QLEDs have achieved T_50_ and T_95_ lifetimes at 100 cd·m^−2^ exceeding 10^6^ h and 5 × 10^5^ h, respectively, meeting the operational requirements for commercial displays. However, blue QLEDs lag substantially behind, with reported T_50_ lifetimes often an order of magnitude shorter than their red and green counterparts [190]. This color-dependent stability gap, commonly originating from hole accumulation and oxidation at the blue CQD/HIL interfaces, remains the critical obstacle to the commercialization of full-color QLEDs.

While efficiency and lifetime remain central concerns, substantial effort has also targeted color quality, as evaluated by the color rendering index (CRI) and color correlated temperature (CCT) and patterning resolution, a prerequisite for high-pixel-density display applications.

In white QLEDs (WQLEDs) that combine several CQD emitters in one film, the emission spectrum is not simply the sum of the individual emitters. An exciton on one CQD can move to a neighbor without any charge transfer. Owing to their broad absorption, higher-energy (blue/green) CQDs can transfer excitons non-radiatively to nearby lower-energy (red) CQDs through dipole-dipole coupling without involving net charge transport. This process is known as Förster resonance energy transfer (FRET). The FRET rate between a donor and an acceptor separated by distance *d* is given by:

$$k_{F}\left(d\right)=\frac{1}{\tau_{D}}{\left(\frac{R_{0}}{d}\right)}^{6}$$

where the *τ_D_* is the donor lifetime in the absence of the acceptor, *R*_0_ is the Förster radius. In composite emissive layers and color-conversion films, this spectral overlap-driven exciton funneling skews the emission spectrum toward the red region and often leads to efficiency degradation. Consequently, traditional strategies seek to suppress FRET by spatially isolating emitters using stacked, patterned, or superparticle-based architectures [191,192,193]. Conversely, recent work has begun to use CQD-to-CQD FRET as a deliberate design parameter rather than only a loss. In R/G/B QD PMMA composite emissive layers, the polymer content sets the interdot spacing and hence the FRET rate, which tunes the electroluminescence from greenish to reddish white at a fixed RGB ratio and constant bias, with nearly pure white emission close to CIE (0.33, 0.33) obtained without significant loss of electrical properties [194]. In white QLEDs with a single emissive layer built from heavy-metal-free R/G/B QDs, FRET between the mixed QDs was compensated by testing different R/G/B mixing ratios and selecting the optimal one, which improved both the light quality and the device efficiency, giving a peak EQE of 4.9% [195]. Interference color filters then generated the individual R, G and B colors from this single white light source, and the display reached a color gamut of 113% NTSC.

However, despite its unique utility in color tuning, FRET in composite emissive layers is overwhelmingly treated as a loss mechanism. Because non-radiative energy transfer redirects excitons toward species with lower bandgaps or higher non-radiative recombination rates, it inherently depresses the overall EQE and disrupts charge balance in the device. Furthermore, quantitative theoretical and simulation models of FRET dynamics within dense, multi-species WQLED thin films remain scarce. Most existing models rely on idealized point-dipole approximations that fail to fully capture complex multi-body interactions, random spatial packing, and electric-field-induced exciton migration under bias. Nevertheless, computational frameworks such as kinetic Monte Carlo (KMC) simulation and rate-equation modeling offer a promising route to describing FRET quantitatively [196]. By tracking exciton hopping between dots, with transfer rates set by the interdot spacing and the donor-acceptor spectral overlap, these methods can predict where excitation is transferred and where it is lost. Such predictions would allow FRET to be treated as a design parameter in the choice of R/G/B ratio and interdot spacing, and no longer as an uncontrolled loss channel, which is essential for next-generation high-efficiency WQLEDs.

#### 4.3.2. Simulation Modeling of QLEDs

Compared with QDSCs and QDPDs, simulation studies of QLEDs remain relatively scarce because their operation requires the coupled description of charge injection and transport, exciton formation and radiative and nonradiative recombination, making the electroluminescence process more difficult to capture within conventional TCAD frameworks (Table 3). While TCAD is well established for modeling carrier transport in crystalline semiconductors through drift-diffusion equations, QLEDs involve additional mechanisms associated with quantum-confined CQDs, including hopping transport, exciton formation, trap-assisted processes and strong interfacial effects arising from ligand passivation. Furthermore, many of the material parameters required for TCAD simulations, including carrier mobility, trap density and interface properties, are not well defined and depend strongly on quantum dot synthesis and device fabrication, limiting the application of conventional TCAD to QLED models. Consequently, most simulation studies rely on specialized electrical, optical, or atomistic modeling techniques tailored to specific aspects of device operation.

The majority of QLED simulation studies used a combination of TMM [197,198], FDTD [199,200,201,202], DFT and rate-equation models [203] to provide comprehensive descriptions of charge transport, exciton dynamics and light extraction in QLEDs (Table 3). Among these, TMM calculates the optical field distribution under the assumption that the QLED layers are optically isotropic and homogeneous, with the Poynting vectors used to evaluate the resulting optical power flow [204]. Because TMM assumes planar, optically homogeneous layers, it is primarily suited to idealized device structures and does not fully capture morphology-dependent optical interactions in practical QLEDs. It is therefore often complemented by other optical simulation methods, with FDTD employed to account for the morphology of constituent layers [36,205].

**Table 3:** Comparison of simulation methods and reported performance metrics for QLED studies. The best reported values for each performance metric are marked with (*). In the EQE column, R, G, and B denote single-color red, green, and blue QLEDs, respectively, and W denotes WQLEDs.

Ref.	Public Year	Tool	Modeling Focus	EQE (%)
[206]	2026	TCAD	Black-matrix sub-pixel isolation for near-eye displays	R: 7
[203]	2026	rate-equation	I-V characteristics and ideality-factor modeling	R: 12–15; G: 10–12; B: 8–10
[207]	2025	LAOSS (Fluxim)	MLA density/radius, dipole orientation	G: 14.69; R: 19.87
[198]	2025	TMM + discrete grid search	Angular chromaticity, CCT optimization	R: -
[208]	2025	TCAD + compact model + WKB	Ionic-liquid-doped QD emissive layer	R: 17.0, 20.5 (+PMMA)
[200]	2025	DFT + FDTD	Long-range-ordered blue QD microstructure arrays	B: 24.1 (*)
[209]	2024	Setfos	Top-emitting cavity/refractive-index engineering	G: 29.2 (*)
[210]	2024	Drift-diffusion + extraction fit	Combinatorial exciton-recombination parameterization	R: 9.8; G: 6.8; B: 2.3
[211]	2023	COMSOL Multiphysics	Novel TE-QLED RaDiNa structure	R: enhanced 60%
[197]	2023	Dipole model + TMM	Electrode/outcoupling optimization (IZO vs. ITO, MLA)	R: 33.2–37.5; G: 18.8; B: 14.4
[212]	2023	Active learning + DFT + MD	HTM materials screening	-
[199]	2022	2D/3D FDTD	Nanopillar + grating LEE structure	B: 29.7; R: 25.9
[213]	2022	Setfos + experiment	Substrate engineering (glass vs. Si/SiO_2_)	R: 20.7
[214]	2022	Modified Langevin rate-equation model	Sequential e^−^/h^+^ injection order; trion (eeh/ehh) pathways	-
[193]	2022	ML + charge-transport simulation	white QLED design	W: ~1 per pixel
[202]	2020	FDTD	Outer-surface multiscale nanostructures	G: 12.29–17.94

##### Outcoupling Enhanced QLEDs

Simulation studies of QLEDs have primarily focused on improving the EQE, as poor OCE represents a dominant loss channel in BE-QLEDs. Conventionally, only 20~30% of initially generated photons escape into free space, with the remainder being trapped in waveguide, substrate and surface plasmon-polariton modes [215,216,217]. Several studies have investigated the optimization of structural parameters to enhance EQE. Lee et al. combined classical-dipole optical simulations with experiments to guide substrate selection and optimize layer thicknesses before device fabrication. Optimal red QLEDs achieved an EQE of 20.7%, while the optimized optical design enabled a luminance of 3.3 × 10^6^ cd·m^−2^, a current efficiency of 75.6 cd·A^−1^ and an extrapolated T_50_ lifetime of approximately 1.25 × 10^8^ h at 100 cd×m^−2^ [213]. Li et al. combined a classical oscillating-dipole model with TMM to optimize electrode material and thickness [197]. By replacing the conventional ITO electrode with an optimized indium zinc oxide (IZO) electrode possessing a higher refractive index, the EQE in red QLEDs reached 33.2%, corresponding to a 1.4-fold improvement compared to the QLEDs with a conventional ITO electrode.

**Figure 6:**
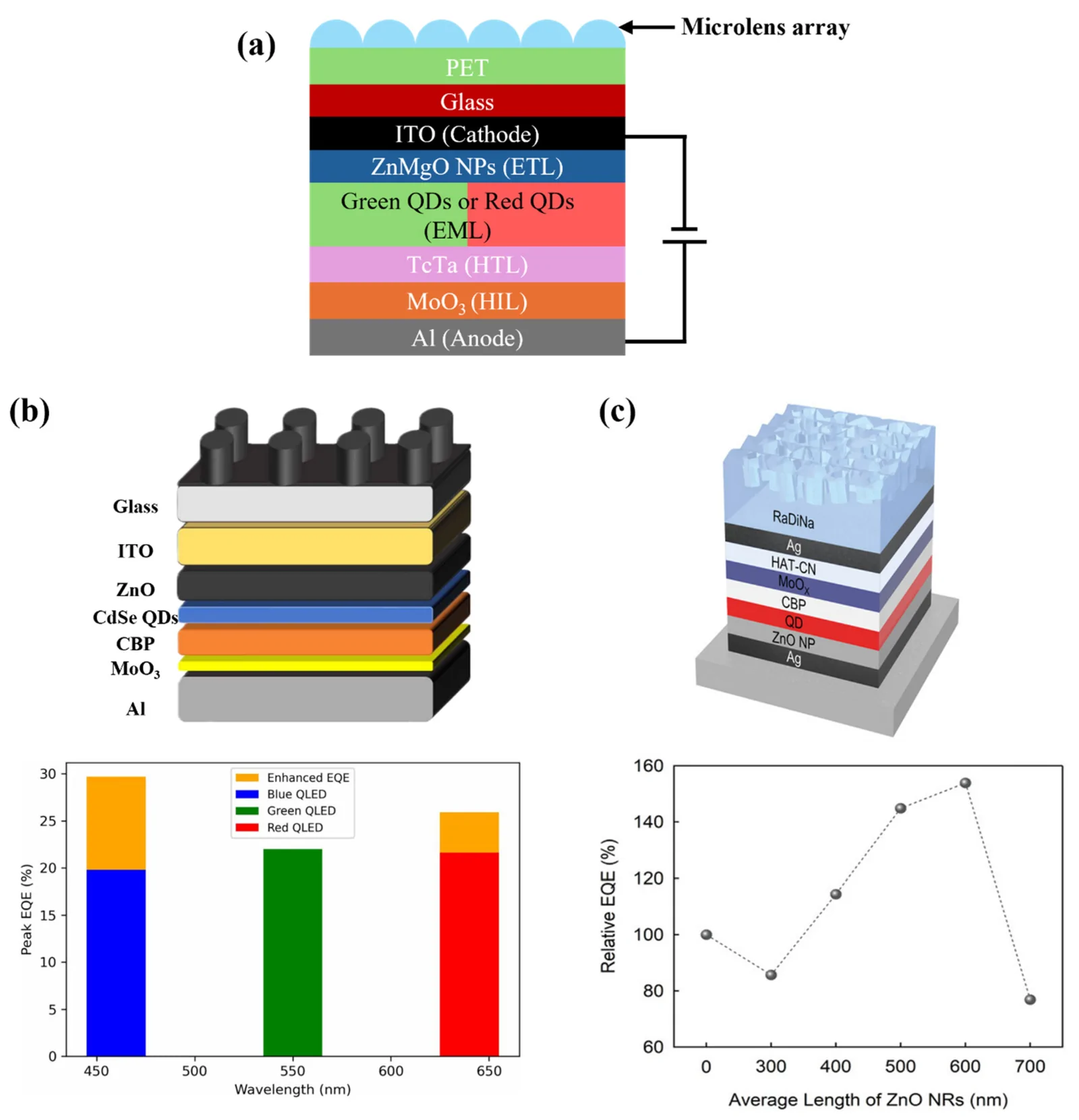
Novel outcoupling structures: (**a**) device architecture in QLEDs with a microlens array [207], (**b**) device architecture and peak EQE in nanopillar QLEDs (reproduced from Ref. [199] under the Creative Commons Attribution 4.0 International (CC BY 4.0) license). (**c**) Device architecture and relative EQE in RaDiNa-attached TE-QLED structure (reproduced with permission [211]. Copyright 2023 John Wiley and Sons).

Layering a high-index plastic substrate with a microlens array (MLAs) pushed the EQE in red QLEDs to a record 37.5%. The same optical framework projected the theoretical EQE ceiling for red, green and blue QLEDs at 35.4~36.5%, 24.8~34.0% and 25.1~35.8%, respectively, in the absence of any outcoupling structure. Simulation studies of MLAs for improving OCE date back to hemispherical MLAs fabricated on a flexible substrate originally demonstrated for inverted QLEDs [218]. Recently, Park et al. employed the commercial Large-Area Organic Semiconductor Simulation (LAOSS) optical simulation package (Fluxim) to optimize MLA film density and lens radius [207]. These simulations suggest that the outcoupling benefit of an MLA is strongly coupled to the emitter’s transition dipole moment orientation, with more vertically oriented dipoles extracting more efficiently through the MLA (Figure 6a). Their green and red QLEDs exhibit outstanding EQEs of 14.69% and 19.87%, with luminance enhancements of 1.51 times and 1.28 times at 5 V, respectively, which represent the highest experimentally reported EQEs for QLEDs incorporating MLAs.

Othman et al. proposed an addition of ZnO nanopillars on top of the glass layer to enhance the OCE using 2D and 3D FDTD [199]. Systematic tuning of nanopillar height, radius, pitch and material yielded OCE enhancements of 50% at 460 nm and 20% at 640 nm relative to a planar reference stack, translating into theoretical EQE ceilings of 29.7% for blue QLED and 25.9% for red QLED (Figure 6b). Another outcoupling strategy was recently introduced by Wang et al., where nanostructures are attached to the outer surface of the glass substrate, improving green EQE and luminance from 12.29% to 17.94% and from 122,400 to 178,700 cd·m^−2^, respectively, with a peak current efficiency of 88.3 cd·A^−1^ [202]. Moreover, a novel light extraction structure, called the randomly disassembled nanostructure (RaDiNa), was introduced and simulated using COMSOL, achieving a 60% enhancement in EQE relative to the reference device (Figure 6c) [211].

These three structural approaches, including RaDiNa, MLA and ZnO nanopillar structures, are implemented as external layers on top of the QLED stack, improving light extraction without altering the internal device architecture. These studies model the CQD emissive layer as a fixed dipole source and focus on optical and geometrical parameters, while charge injection and carrier recombination processes governing IQE and EQE are not considered. The following studies incorporate these electrical processes to address the internal operation of QLEDs.

##### Charge Injection and Internal Quantum Efficiency Enhanced QLEDs

Poor hole injection is one of the major bottlenecks limiting QLED performance, as it is the primary cause of charge imbalance. Abroshan et al. directly screened the ETL and HTL materials using an active learning workflow coupled with high-throughput DFT, evaluating approximately 8600 candidates of transport layers from an initial chemical space of more than two million molecular structures [212]. The most promising candidates were further evaluated using molecular dynamics simulations of amorphous thin films, Marcus’s theory-based estimates of hopping rates, random walk simulations and glass transition temperature predictions, with the overall analysis targeting the charge injection imbalance responsible for suppressed IQE.

Another line of recent work has focused directly on recombination pathways. Kim et al. combined drift-diffusion transport with an ABC-type recombination model (SRH rate A, bimolecular/Langevin rate B, Auger rate C), together with photon extraction efficiency parameters. All parameters were fitted to experimental data and the resulting framework reproduced the experimental EQE roll-off with the onset of efficiency-droop attributed to a dominant recombination channel [210]. Yu et al. developed a modified Langevin rate-equation model to investigate the electronic and excitonic dynamics of exciton formation, explicitly distinguishing the negative trionand positive trion recombination channels that dominate Auger quenching in charged QDs [214]. They simulated three transport layers, ZnO, PEDOT: PSS and TiO_2_, which induce negative, positive and intermediate QD charge states, respectively. The results showed that electron injection before hole injection is required for efficient QLED operation because the resulting negative trion exhibits a lower intrinsic Auger recombination rate than the positive trion.

##### Light Quality Metrics and Pixel Resolution in QLEDs

The last group of QLEDs modeling studies is not primarily to maximize peak EQE, but to optimize other performance dimensions relevant to display and lighting applications, including light quality and pixel resolution. These studies often accept modest EQE as a deliberate trade-off.

Li et al. used Setfos optical-mode simulation with a top-emitting cavity strategy, achieves a EQE of 29.2% for green QLEDs, luminance exceeding 1.6 × 10^6^ cd·m^−2^, current efficiency of 204.2 cd·A^−1^, T_95_ lifetime of 15,600 h at 1000 cd·m^−2^ and an angular color shift of only Δ*u*′*v*′ = 0.0052 from 0° to 60°, demonstrating that single-mode microcavities can be engineered to preserve color uniformity in QLEDs because of their narrower emission linewidth [209]. Jung et al. generalized this computational problem using TMM combined with a discrete grid-search algorithm to map how multilayer thickness configurations govern angular chromaticity and CCT in both single-color and white-lighting QLEDs, achieving angular chromaticity differences below 0.02 and CCT stability within 6500 ± 200 K over a wide angular range [198].

At the system level, the white-lighting computational-design framework conducted by Samarakoon et al. integrates system-level color optimization with device-level charge-transport simulation [193]. A machine learning Nelder-Mead algorithm was used to identify the optimal QD emission wavelengths and color-weighting factors for high-quality white light, while an electric field-dependent charge injection model predicted device performance using experimentally derived material parameters. This two-stage framework achieved a theoretical maximum CRI of 97 and an experimentally validated CRI of 92 across a wide CCT range of 1612~8903 K, with an EQE of each pixel that remained low at ~1%. Despite the low efficiency, the close agreement between predicted and measured color quality shows that such a workflow can guide the selection of emitters and their mixing ratios before fabrication. Extending the framework to include efficiency-limiting processes such as FRET and Auger recombination is therefore a promising route toward WLEDs that combine high color quality with high EQE.

Recent advances in high-resolution QLEDs for near-eye displays have been supported by TCAD simulations evaluating a black-matrix insulating barrier that electrically and optically isolates adjacent sub-pixels by preventing direct ETL-HTL contact in non-emissive regions [206]. The simulated device reached 2540 pixels-per-inch (PPI) resolution with a luminance of 7503 cd·m^−2^ and an EQE of only 7%. Following a similar objective, the blue QD microstructure array was designed using combined DFT-based optimization calculations with FDTD optical simulation to design an aromatic-enhanced capillary-bridge confinement strategy for patterned blue QLED arrays [200]. The resulting device reached a peak EQE of 24.1% and luminance of 1.0 × 10^5^ cd·m^−2^ at a resolution exceeding 500 PPI, which is 1.8 to 5 times higher than the corresponding disordered-array device with the ordering strategy simultaneously suppressing defect-mediated non-radiative loss and enabling finer pixel patterning.

Another study of quantum dot electrolyte LEDs proposed a new device category incorporating an ionic liquid into the CQD emissive layer using 2D TCAD with an analytical compact model, WKB-tunneling calculations and extensive experimental validation to resolve electric-field redistribution, energy-band bending, charge injection and ion-accumulation dynamics [208]. This study also introduces PMMA into the ionic liquid/CQD layer which raises EQE from 17.0% to 20.5% and extended the T_95_ lifetime to ~3.74 × 10^5^ h at 100 cd·m^−2^, illustrating TCAD’s diagnostic value for a fundamentally different charge-transport regime.

Overall, the theoretical EQE limits for red, green and blue QLEDs have been investigated to be 35.4~36.5%, 24.8~34.0% and 25.1~35.8%, respectively. Various studies employed simulations to optimize device architecture, outcoupling structures and ETL/HTL materials, leading to an experimentally confirmed EQE of 24.1% for blue QLEDs [219]. The highest reported EQE is 38.2% for red QLEDs, reaching 44.5% with the incorporation of a scattering layer on the IZO electrode [193], whereas that for green QLEDs exceeds 29.2% [209].

## 5. Conclusions and Outlook

Over the past decade, CQDs have progressed from laboratory curiosity to a multifunctional platform for optoelectronics and theory has advanced in parallel, from atomistic descriptions of isolated nanocrystals to device simulation of overall architectures. This review has traced the recent progress of CQD-based optoelectronic devices, covering material synthesis and characterization, working principles and device architectures across three major applications: QDSCs, QDPDs and QLEDs.

Across all three device classes, theoretical CQD frameworks have transitioned from a tool for interpreting experimental observations to predictive approaches for guiding material design and device optimization. In QDSCs, simulation-guided design has enabled quantitative understanding of charge transport, interfacial losses and advanced architectures beyond conventional efficiency limits. In QDPDs, multiscale simulations have provided insights into carrier multiplication, current noise suppression and photoresponse optimization across various device architectures. In QLEDs, optical and exciton-dynamic models based on TMM, FDTD and rate-equation approaches have guided light extraction and efficiency optimization toward theoretical limits.

Nevertheless, this shift is far from complete, and the remaining gaps concern both the scope and the use of current simulations. In terms of scope, complex architectures based on CQDs, including tandem structures, perovskite/CQD hybrids and emerging lead-free CQD systems, remain insufficiently explored by simulation despite their potential for further performance enhancement. Moreover, QLED simulations have primarily focused on efficiency optimization, whereas the analysis of operational lifetime and simultaneous optimization between quantum efficiency and light quality remain largely unexplored. In terms of use, most studies still employ simulation as a supporting tool for analyzing or interpreting experimental results rather than as a predictive framework, and many lack sufficient experimental validation, which limits the reliability and practical applicability of their predictions.

These gaps are most consequential for the practical bottlenecks that restrict the industrialization of CQD optoelectronics, which remain to be addressed from both experimental and theoretical perspectives. The first is stability, which is governed by the CQD film (Section 3.4). Current simulations treat small dots, one stressor and static binding energies, so they explain degradation but cannot forecast it. Machine-learned potentials trained on DFT data could extend MD to realistic dot sizes, ligand coverages and temperatures, and the resulting binding and desorption energies could enter device models as time-dependent trap rates, so that lifetime is predicted from surface chemistry. Second is large-area fabrication, which faces the same environmental sensitivity for longer processing times, together with thickness and packing non-uniformity that atomistic studies and idealized device models both ignore. Linking surface-chemistry simulations, film-formation models and large-area device models, with ML surrogates to make the chain affordable, would allow processing windows and coating conditions to be chosen before fabrication. White-light emission raises a different but related difficulty. For WLEDs, FRET is a central difficulty. Energy transfer from blue and green CQDs to red CQDs shifts the color balance and lowers device efficiency, and it is hard to predict because current computational frameworks absorb it into fitted efficiency parameters, so its effect on color and efficiency only becomes visible after a device has been made. Controlling FRET is therefore essential for improving WLEDs. To achieve this, simulation must treat energy transfer explicitly. A coupled model of charge transport, energy transfer and recombination, with parameters such as the transfer radius inferred from time-resolved data by ML would make the loss quantifiable at the design stage. Such a model would allow emitter ratios, spacing and driving conditions to be optimized together, so that energy transfer is suppressed where it is a loss and exploited where it can tune the color, and it would allow the color drift with aging to be predicted.

**Figure 7:**
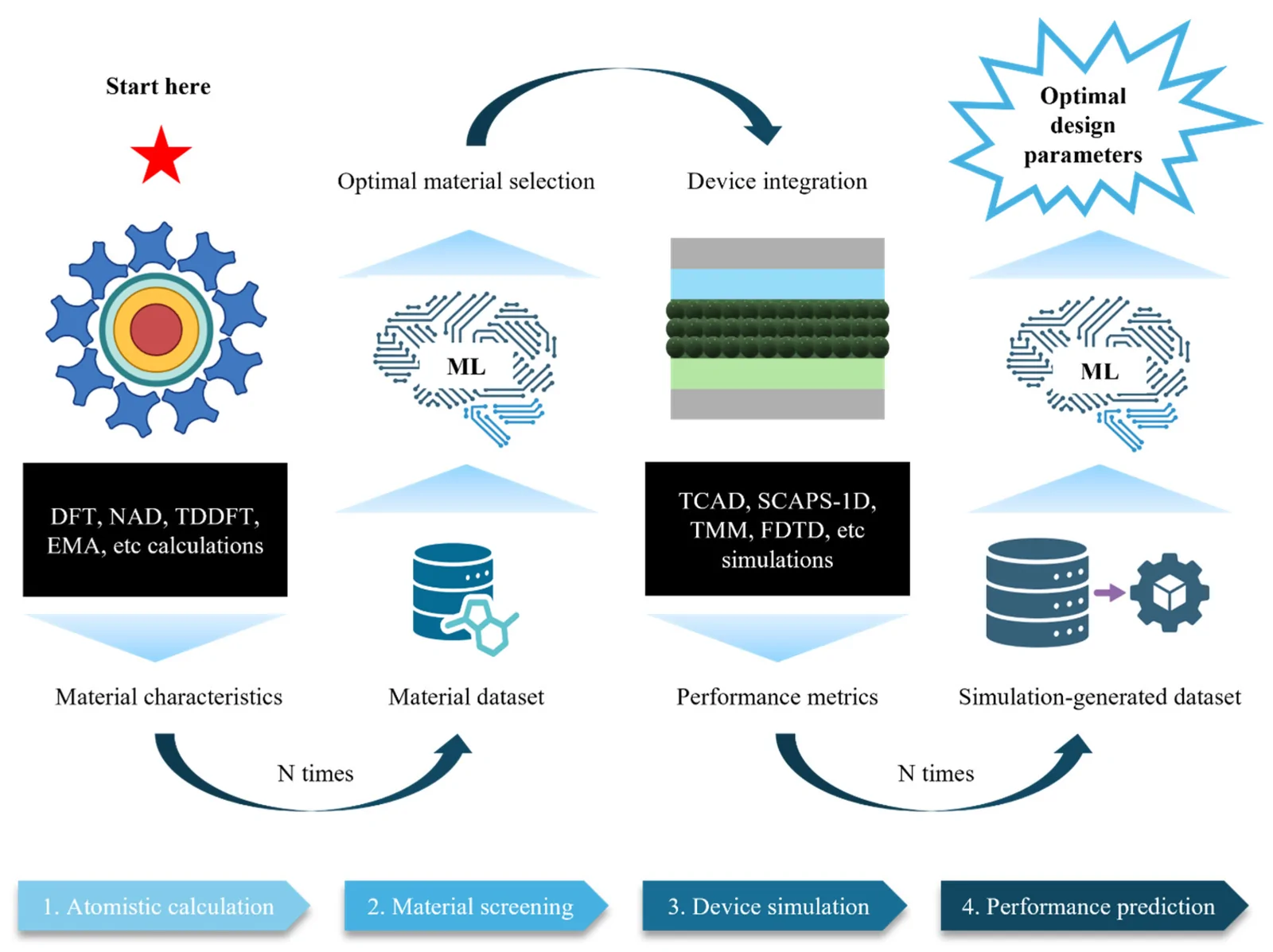
Envisioned multiscale integrated computational framework for the future rational design of CQD-based optoelectronic devices, combining atomistic calculation, machine learning and device simulation to accelerate next-generation performance discovery.

Addressing these challenges will require simulation to move from explaining experimental results to guiding design. The three bottlenecks are linked through the CQD surface, which sets film stability, processing tolerance and the recombination losses that shape white emission, so they are best tackled together rather than one by one. Machine learning (ML)-assisted simulation, with its capability to handle high-dimensional and multivariable problems, can accelerate simulations and enable efficient optimization of CQD material properties and device architectures across a broad parameter space. Such an approach offers a promising route toward improving stability, white-light performance and large-area fabrication. Future advances toward the commercialization of CQD optoelectronic applications will require multiscale computational frameworks that bridge atomistic material understanding with device performance prediction (Figure 7). Combining computational material screening with device simulations will enable more efficient exploration of novel CQD materials and optimized device architectures, while ML approaches trained on simulation-generated datasets may further accelerate the identification of optimal design parameters. Indeed, ML has already played an important role in optoelectronic sensing, particularly in computational spectrometry, where electrically programmable band profiles are combined with neural-network decoders to enrich spectral encoding within a single detector. For example, a fully connected neural network trained on measured photocurrent–spectrum pairs has been used to decode the bias-dependent photocurrents of a dual-bottom-gate black phosphorus photodetector, enabling reconstruction of single-peak spectra over 1200–1700 nm and of one- to eight-peak spectra within a calibrated discrete wavelength basis [220]. Similarly, in a WSe_2_ photonic memristor, whose nonlinear bias- and power-dependent photoresponse cannot be handled by a conventional responsivity-matrix approach, a neural network trained on measured data, combined with a nonlinear-regression preprocessing step, reconstructed the incident spectrum [221]. In both cases, however, ML serves as a post-fabrication decoder where the device is first fabricated and characterized experimentally and a network trained on device-specific measurements then solves the inverse problem from photocurrent to spectrum. The role of ML in CQD optoelectronics is expected to be different. In CQD solids, the spectral response is largely determined during synthesis by dot size and composition, whereas charge transport is governed by hopping, trap states and ligand-dependent inter-dot coupling. Because the dot size distribution, surface ligands and trap density vary between separate syntheses and between individual films, nominally identical CQD devices can exhibit noticeably different photoresponses and a decoder trained on one device is unlikely to transfer to another without recalibration. For CQDs, the more valuable role of ML therefore lies upstream of fabrication, where it can act as a surrogate for computationally expensive atomistic and device simulations and as a tool for materials screening and inverse design of device architectures. Training on simulation-generated datasets, calibrated against limited experimental data, would provide physically grounded priors, reduce the dependence on large, measured datasets and prioritize the CQD architectures and design strategies that are most promising for experimental implementation. Ultimately, the integration of multiscale modeling and machine learning holds strong potential to enable predictive discovery and rational design of CQD materials and devices, accelerating the development of next-generation optoelectronic technologies.

## Figures and Tables

**Table 1:** Summary of recent simulation studies on QDSCs. The best reported value for PCE is marked with (*).

Ref.	Public Year	Device Architecture	Tool(s)	PCE (%)	Exp-Validation
[132]	2026	FTO/TiO_2_/Ag_2_S CQD/PEDOT: PSS/Ag	SCAPS-1D	16.86	No
[133]	2026	ITO/SnO_2_/BaTiO_3_ (polarized)/AgBiS_2_-ZnS-ZnSe/Li: NiO_x_/Au	SCAPS-1D	28.43	No
[134]	2025	FTO/Ag_2_S/Pb-QD/Cu_2_O	SCAPS-1D	32.9 (*)	No
[135]	2024	FTO/ZnS/TiO_2_/PbS-TBAI/PbS-EDT	SCAPS-1D	17.12	No
[136]	2024	ITO/ZnO/InAs/MoO_x_/Au	TMM	2	Yes
[137]	2024	ITO/ZnO/PbS-TBAI/PbS-EDT	TMM + SCAPS-1D	16.48	Yes
[138]	2023	Ag/MoO_3_/Organic layer/PbS CQD/EIL/ZnO/ITO	SCAPS-1D	14.1	Yes
[139]	2023	ITO/ETL (WS_2_/WO_3_)/CdS/Sb_2_Se_3_/PbS/Au	SCAPS-1D	20.6	No
[140]	2023	ITO/TiO_2_/PbS-TBAI/MoO_3_/Au	SCAPS-1D	16.43	No
[141]	2021	ITO/TiO_2_/PbS-TBAI/PbS-EDT/Au	SCAPS-1D	13.14	No
[142]	2021	ITO/TiO_2_/Perovskite/Spiro-OMeTAD//MZO/PbS-TBAI/PbS-EDT/Au	SCAPS-1D	23.36	No
[143]	2021	ITO/ZnO/PbS/Polymer/MoO_3_/Au	SCAPS-1D	13.74	Yes
[144]	2020	ITO/TiO_2_ or ZnO/PbS-TBAI/PbS-EDT/Au	SCAPS-1D	15	No
[145]	2020	a-Si: H/PbS monolithic tandem	TCAD	12	No
[146]	2019	ITO/ZnO/PbS/Au	TMM + SCAPS-1D	11.1	Yes

**Table 2:** Comparison of simulation-based QDPD studies. The best reported values for each performance metric are marked with (*). D* values are annotated NSD (from measured noise spectral density) or SNL (shot-noise-limited method) to allow direct comparison across studies.

Ref.	Public Year	Device Architecture	Tool(s)	Range (nm)	*R* (A/W)	D* (Jones)	EQE (%)
[166]	2026	p-i-n SACM APD ITO/PbS-EDT layer/PbS/PbS charge layer/i-ZnO/n-ZnO/Au	TCAD	SWIR	-	1.15 × 10^12^ (NSD)	130
[32]	2026	ITO/PEDOT:PSS/PbSe:PVK or P3HT/PCBM/Ag	DFT + TCAD	NIR 980	17.89	2.28 × 10^14^ (SNL)	-
[170]	2026	CdSe/ZnS-sensitized MoTe_2_	COMSOL + TCAD + DFT	Vis 520 (630)	0.0013 (0.002)	9.4 × 10^8^ (1.34 × 10^9^) (SNL)	0.31 (0.39)
[171]	2025	p-i-n ITO/ZnO/PbS-halide/PbS-EDT/Au	SCAPS-1D	SWIR 1300	0.65	5.73 × 10^12^ (SNL)	66.5
[172]	2025	ITO/ZnO/PbS/Au-NPs/PbS/P3HT/Ag	COMSOL	NIR 980	1088.86	1.2 × 10^14^ (SNL)	138,006 (*)
[173]	2025	InSb/ZnSe core/shell QD array	NEGF	MIR 4000 (3000)	3.3 (6)	0.9 × 10^11^ (1.5 × 10^11^) (SNL)	-
[40]	2025	p-i-n APD Au/PbS/ZnO/ITO	TCAD + DFT + COMSOL	IR (940)	-	1.4 × 10^14^ (NSD)	239
[174]	2024	ITO/ZnO/CsPbI_3_:PbSe/CuSCN/Au	TCAD	IR (980)	9.29	3.17 × 10^14^ (SNL)	-
[175]	2023	p-i-n ITO/NiO_x_/EDT-PbS/PbS/TFCA-PbS/Ag	Setfos (rate-equation)	NIR (1350)	-	-	68.5
[176]	2023	Cr/Al_2_O_3_/IGZO/CdSe QD (TFT-gated)	TCAD	Vis	21,200 (*)	2.74 × 10^16^ (*) (SNL)	-
[177]	2022	ITO/ZnO/PbSe:CsPbBr_1.5_I_1.5_/P3HT/Au	TCAD	Vis-IR	6.16	5.96 × 10^13^ (SNL)	17.15 (405 nm) 18 (532 nm)
[34]	2022	ITO/As_2_Se_3_/a-Se/CdSe/CeO_2_/ITO	TCAD	Vis (405–656)	<0.2	0.5–5 × 10^12^ (NSD)	55 (430 nm)

## Data Availability

The authors declare that the primary data underpinning the findings of this study are available within the article. Additional data are available from the authors upon request.
